# E-Nose and HS-SPME-GC-MS unveiling the scent signature of *Ligusticum chuanxiong* and its medicinal relatives

**DOI:** 10.3389/fpls.2025.1476810

**Published:** 2025-03-10

**Authors:** Wanjing Xu, Chao Zhang, Rong Xu, Juan Yang, Yijuan Kong, Li Liu, Shan Tao, Yu Wu, Hailang Liao, Changqing Mao, Zhengjun Xu, Fang Peng

**Affiliations:** ^1^ Industial Crop Research Institute, Sichuan Academy of Agricultural Sciences, Chengdu, Sichuan, China; ^2^ Crop Ecophysiology and Cultivation Key Laboratory of Sichuan Province, Sichuan Agricultural University, Chengdu, Sichuan, China; ^3^ State Key Laboratory for Quality Ensurance and Sustainable Use of Dao-di Herbs, Institute of Medicinal Plant Development, Chinese Academy of Medical Sciences, Beijing, China

**Keywords:** *Ligusticum chuanxiong* Hort., E-nose, odor profiles, headspace solid phase microextraction, gas chromatography-mass spectrometry, volatile components, affinities

## Abstract

**Introduction:**

To explore the origin and evolution of Ligusticum Chuanxiong, we conducted a component analysis of Ligusticum Chuanxiong and its medicinal relatives.

**Methods:**

This study encompassed seven species from various origins, including Chuanxiong (*Ligusticum chuanxiong* Hort.), Gansu Chuanxiong (*Ligusticum chuanxiong* cv. Gansu), Yunnan Chuanxiong (*Ligusticum chuanxiong* cv. Yunnan), Japanese Chuanxiong (*Cnidium officinale* Makino), Fuxiong (*Ligusticum sinense* ‘Fuxiong’), Gaoben (*Ligusticum sinense*), and Liaogaoben (*Ligusticum jeholense*), comprising 27 distinct materials. We employed headspace solid-phase microextraction-gas chromatography-mass spectrometry (HS-SPME-GC-MS) to identify various odor profiles from these species using electronic nose technology (E-nose). The method effectively identified volatile constituents in the leaves of these seven species.

**Results:**

Results indicated that odor differences between *L. chuanxiong* and its medicinal relatives were predominantly observed in sensors W1W and W1S. Linear discriminant factor analysis (LDA) successfully distinguished five of the relatives; however, *L. chuanxiong* and *L. sinense* exhibited high odor similarity, limiting complete differentiation in some samples. HS-SPME-GC-MS identified a total of 118 volatile constituents, with eight differential volatiles identified: trans-Neocnidilide, β-Caryophyllene, β-Selinene, 5-Pentylcyclohexa-1,3-diene, (E)-Ligustilide, Butylphthalide, Neophytadiene, and Senkyunolide. Hierarchical cluster analysis (HCA) grouped *L. chuanxiong*, *L. sinense*, *L. jeholense*, and *L. chuanxiong* cv. Gansu together, highlighting the close relationship between *L. chuanxiong* and *L. sinense*. Joint analysis revealed a significant positive correlation between sensor W1W and the differential volatile component β-Caryophyllene, suggesting its potential for distinguishing closely related species.

**Discussion:**

This study provides a foundational understanding of volatile components in the leaves of *L. chuanxiong* and its medicinal relatives using E-nose combined with HS-SPME-GC-MS, contributing to the discussion on their interspecific odor characteristics and relationships.

## 1 Introduction

The cultivation of medicinal plants boasts a remarkable history spanning over 5,000 years, and the story of human domestication of these essential crops has long captivated our curiosity. Researchers have tirelessly employed a diverse array of techniques and methodologies to unravel the evolutionary trajectories of these plants, yielding a wealth of groundbreaking findings (Teixidor-Toneu et al., 2018; Yin et al., 2023; Yang et al., 2023). These investigations have not only illuminated our understanding of the origins and evolutionary histories of these cultivated species, paving the way for enhanced conservation and utilization of their genetic resources, but have also enriched our comprehension of the evolutionary narrative of human pharmaceutical civilization. Concurrently, the theoretical and methodological frameworks established through the study of the domestication origins and evolution of cultivated medicinal plants have significantly propelled the advancement of medicinal plant phylogeny (Hao et al., 2015; Youssef et al., 2023). *Ligusticum chuanxiong* (CX), part of the genus Ligusticum in the family Umbelliferae, is among the oldest cultivated and most popular medicinal plants globally. Cultivated since the Qin and Han Dynasties (Zheng et al., 2021), the rotation of CX with rice has now become an exemplary food and herb rotation model in China. CX has profoundly influenced the culture, health, medicine, and trade of the Chinese people and played an important role in the daily lives of many in Asia and worldwide (Li et al., 2012). CX, included in the Chinese Pharmacopoeia as Chuanxiong Rhizoma, is widely used in Japan, Taiwan, and Korea to promote blood circulation and eliminate stagnation, with Sichuan being its main production area (Ran et al., 2011). Its young leaves are commonly used as edible materials, such as in salad dressings, stewed vegetables, and other dishes, and have a positive effect on treating dizziness (Chen et al., 2018).

Every cultivated plant has a wild ancestor, but no wild resource (wild type) of CX has been found to date. Therefore, exploring its relationship with its relatives is the most common method to trace its evolutionary history. The primary medicinal relatives of CX are *L. sinense* (GB), *L. jeholense* (LGB), *Cnidium officinale* Makino (JCX), *L. chuanxiong* cv. Gansu (GSCX), *L. chuanxiong* cv. Yunnan (YNCX), and *L. sinense* ‘Fuxiong’ (FX). GB and LGB are used as Ligustici Rhizoma et Radix in the Chinese Pharmacopoeia (Chinese Pharmacopoeia Commission, 2020). LGB, an endemic plant species in China, is primarily distributed in the three eastern provinces (Shi et al., 2024). JCX is documented in the Japanese Pharmacopoeia as a traditional medical prescription and has nearly the same medical effects as CX (Ministry of Health and Welfare Press, 2021). GSCX is primarily produced in Guanzhong and Qinchuan, among other regions. YNCX is primarily produced in Dali, Lijiang, and Zhongdian in Yunnan (Zhang et al., 1990). FX, also known as ‘Chaxiong’, originates from Fuzhou, Jiangxi Province, and is primarily used to treat menstrual disorders and postpartum stasis (Jiangxi Food and Drug Administration, 2014). Previous research has explored the relationships among species in the genus Ligusticum using morpho-anatomy (Xing et al., 2024) and gene sequencing (Jigden et al., 2010), but their systematic positions vary with different taxonomic methods. It is evident that previous studies have focused on morphological and molecular aspects, with limited research on chemical composition. The volatile components of CX and its relatives, belonging to the aromatic family of plants, play an important role in their medicinal effects. Therefore, studying their volatile components is of significant interest.

Re-edited the entire paragraph: Odor plays an important role in species identification, especially in identifying closely related species (Oh, 2023; Gonzalez et al., 2022; Ludwiczuk et al., 2013). Modern analytical techniques such as the electronic nose (E-nose) facilitate rapid analysis of odors. The electronic nose (E-nose), a new artificial intelligence olfactory device that can transform sensor signals into electrical signals (Chen et al., 2022). The volatile components in CX rhizomes have been analyzed using E-nose technology to classify and evaluate samples from different origins. (Chen et al., 2013). Volatile constituents are also important in medicinal plant research. Volatile components can be used to understand the medicinal properties of plants or conduct a comprehensive assessment of their biological potential (Hu et al., 2020; Yarazari and Jayaraj, 2022; Aziz et al., 2022). Combined with chemometric analysis, it can also reveal the species differentiation of medicinal plants (Ma et al., 2023). CX produces numerous volatile components that contribute to its medicinal properties. For example, (E)-Ligustilide in CX has great potential for antidepressant and intestinal flora regulation (Zhou et al., 2023), and butylphthalide can promote recovery from sudden deafness (Xiong et al., 2021). The volatile components of the rhizomes of CX have been extensively studied using GC-MS. Establishing GC-MS fingerprints or combining them with the entropy minimization (EM) algorithm can effectively identify volatile components among species (Tang et al., 2022; Yang et al., 2008; Zhang et al., 2007). The headspace solid-phase microextraction (HS-SPME) technique allows automated enrichment of volatiles with high flexibility (Bojko, 2022). Gas chromatography-mass spectrometry (GC-MS) is commonly used to analyze volatile constituents in plants (Song et al., 2023), often combined with HS-SPME to separate and identify complex volatile constituents (Zhang et al., 2018). There is a direct correlation between odor and volatile components. Odor is a subjective sense of smell and is affected by environmental and other factors. Electronic nose technology can quickly and sensitively conduct non-destructive testing of samples to provide overall information on flavor substances, but it is impossible to obtain information on the specific components of the sample. GC-MS can perform accurate qualitative and quantitative analysis of volatile components, but the experimental cost is high, the experimental analysis cycle is long, and online monitoring is difficult. Therefore, the use of electronic nose combined with GC-MS can identify unknown odor types in a short time and provide simple qualitative analysis of volatile components. Scholars have used a combined analysis of odor and volatile components to evaluate the resource diversity of *Platostoma palustre* (Zhong et al., 2024), explore species differentiation of medicinal *Atractylodes* through volatile constituents (Ma et al., 2023), and isolate and identify different *Angelica sinensis* species (Kim et al., 2006).

In this study, the volatile components in 27 samples of Ligusticum chuanxiong and its relatives were compared using headspace solid-phase microextraction gas chromatography-mass spectrometry (HS-SPME-GC-MS) and electronic nose (E-nose) techniques. A volatile composition identification model was developed to distinguish the different species, aiming to elucidate the odor characteristics of CX and provide a reference for clarifying the affinities between CX and its medicinal relatives. In this study, the volatile components of metabolites were chosen for analysis, and for the first time, a systematic comparison of the volatile components in CX leaves and its six medicinal relatives was conducted. This may serve as an important addition to previous studies, providing new perspectives on the systematic position of CX among its relatives within the genus Ligusticum and on the origin of the species.

## 2 Materials and methods

### 2.1 Experimental material

**Figure 1:**
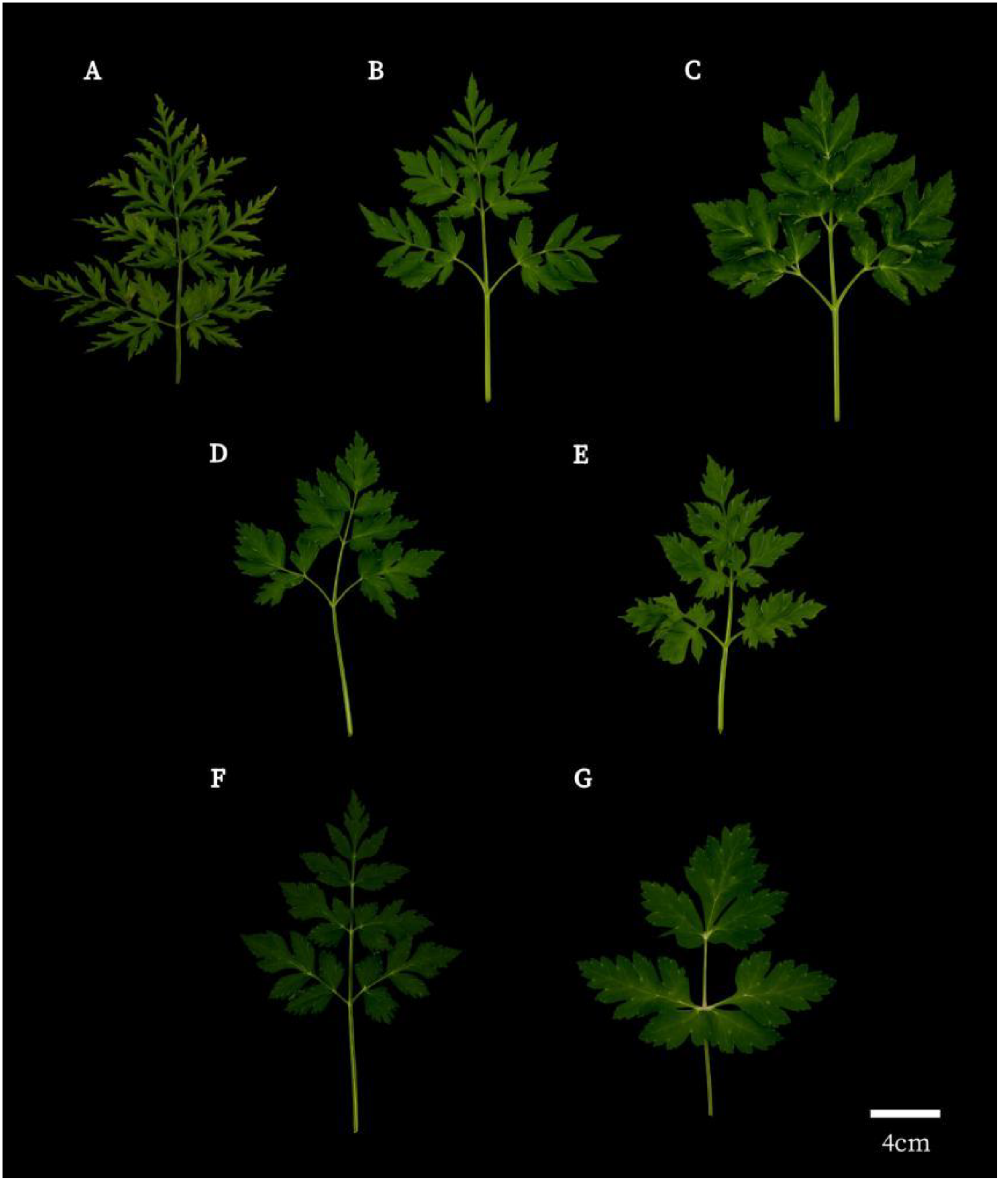
Illustration of the leaf blades of *L*.*chuanxiong* and its relatives. **(A)** *Ligusticum chuanxiong* Hort.; **(B)** *Ligusticum chuanxiong* cv. Gansu; **(C)** *Ligusticum chuanxiong* cv. Yunnan; **(D)** *Cnidium officinale* Makino; **(E)** *Ligusticum sinense* ‘Fuxiong’; **(F)** *Ligusticum sinense*; **(G)** *Ligusticum jeholense*.

A total of 27 medicinal plant samples were gathered from various cultivation areas in China. The samples were identified by researcher Chao Zhang from the Industrial Crop Research Institute of the Sichuan Academy of Agricultural Sciences. The samples comprised *Ligusticum chuanxiong* Hort. (CX), *Ligusticum chuanxiong* cv. Gansu (GSCX), *Ligusticum chuanxiong* cv. Yunnan (YNCX), *Cnidium officinale* Makino (JCX), *Ligusticum sinense* ‘Fuxiong’(FX), *Ligusticum sinense* (GB), and *Ligusticum jeholense* (LGB) (**Figure 1**). To ensure consistency in growth conditions, all collected medicinal plants were cultivated at the planting base of the Economic Crops Research Institute, Sichuan Academy of Agricultural Sciences, under standardized irrigation and fertilization protocols. Samples were collected at harvest time by selecting fully extended, mature leaves. Three biological replicates were gathered for each sample group (**Table 1**).

**Table 1:** Sample information collection.

No.	Herbal Name	Plant Species	Collection Area	Collection Date
1	Chuanxiong	*Ligusticum chuanxiong* Hort.	Qingbaijiang District, Chengdu, Sichuan, China (E 104°28′50.19’’/N 36°18′20.03’’)	2023.6.27
2	Chuanxiong	*Ligusticum chuanxiong* Hort.		2023.6.27
3	Chuanxiong	*Ligusticum chuanxiong* Hort.		2023.6.27
4	Chuanxiong	*Ligusticum chuanxiong* Hort.	Pengzhou, Sichuan, China (E 103°51′54.59’’/N 31°9′32.22’’)	2023.7.31
5	Chuanxiong	*Ligusticum chuanxiong* Hort.		2023.7.31
6	Chuanxiong	*Ligusticum chuanxiong* Hort.	Aba Prefecture, Sichuan, China (E 103°25′25.89’’/N 30°56′1.47’’)	2023.8.1
7	Chuanxiong	*Ligusticum chuanxiong* Hort.		2023.8.1
8	Gansu Chuanxiong	*Ligusticum chuanxiong* cv.Gansu	Huating, Gansu, China (E 106°39’12.67’’/N 35°13’3.22’’)	2023.10.19
9	Gansu Chuanxiong	*Ligusticum chuanxiong* cv.Gansu		2023.10.19
10	Gansu Chuanxiong	*Ligusticum chuanxiong* cv.Gansu		2023.9.21
11	Gansu Chuanxiong	*Ligusticum chuanxiong* cv.Gansu		2023.9.21
12	Yunnan Chuanxiong	*Ligusticum chuanxiong* cv.Yunnan	Dali Bai Autonomous Prefecture, Yunnan, China (E 100°10’35.18’’/N 26°33’36.47’’)	2023.9.25
13	Yunnan Chuanxiong	*Ligusticum chuanxiong* cv.Yunnan		2023.9.25
14	Yunnan Chuanxiong	*Ligusticum chuanxiong* cv.Yunnan		2023.9.25
15	Japanese Chuanxiong	*Cnidium officinale* Makino	Leshan, Sichuan, China (E 102°58′55.96’’/N 29°9′17.16’’)	2023.10.12
16	Japanese Chuanxiong	*Cnidium officinale* Makino		2023.10.12
17	Japanese Chuanxiong	*Cnidium officinale* Makino		2023.10.12
18	Fuxiong	*Ligusticum sinense* ‘Fuxiong’	Jiujiang, Jiangxi, China (E 115°9′53.37’’/N 29°14′31.47’’)	2024.4.28
19	Fuxiong	*Ligusticum sinense* ‘Fuxiong’		2024.4.28
20	Fuxiong	*Ligusticum sinense* ‘Fuxiong’	Ruichang, Jiangxi, China (E 115°19′46.21’’/N 29°26′47.12’’)	2024.4.28
21	Gaoben	*Ligusticum sinense*	Lixian, Sichuan, China (E 103°19′6.42/N 31°33′15.06’’)	2023.11.6
22	Gaoben	*Ligusticum sinense*		2023.11.6
23	Gaoben	*Ligusticum sinense*	Ganzi Prefecture, Sichuan, China (E 102°13’42.49’’/N 29°47’19.86’’)	2023.9.9
24	Gaoben	*Ligusticum sinense*		2023.9.9
25	Liaogaoben	*Ligusticum jeholense*	Jinghai District, Tianjin, China (E 116°49’36.91’’/N 38°57’50.29’’)	2023.7.18
26	Liaogaoben	*Ligusticum jeholense*		2023.7.18
27	Liaogaoben	*Ligusticum jeholense*		2023.7.18

### 2.2 Instruments and equipment

PEN3 Electronic Nose (AIRSENSE, Germany); Gas Chromatography Mass Spectrometer (SHIMADZU GCMS-QP2020), HP-5 MS capillary column (SHIMADZU 0.25 mm x 30 m, 0.25 µm), Solid Phase Microextraction (SPME) fiber holder (Supelco, USA), SPME Fiber Assembly (Supelco 50/30 µm DVB/CAR/PDMS), 15 ml spiral headspace vial (Zhejiang Sainz Scientific Instrument Co., Ltd.), electric blast drying oven (Shanghai Bo Xun Industrial Co., Ltd.).

### 2.3 Experimental methods

#### 2.3.1 Electronic nose measurement

##### 2.3.1.1 Sample treatment

Fresh leaves were collected, washed, and dried in an oven at 45 °C for 24 hours. The dried leaves were then pulverized in a pulverizer and sieved through a 100-mesh sieve. The leaf powder was stored in a sealed plastic bag at -20 °C until analysis. For sample preparation, 0.6 g of the powder were accurately weighed and transferred to a 50 mL centrifuge tube. The tube was sealed with a protective film and allowed to equilibrate at room temperature for 30 minutes prior to analysis. All samples were prepared and analyzed in triplicate.

##### 2.3.1.2 Electronic nose parameterization

Measurements were performed by direct headspace aspiration, with the injection tip inserted directly into a sealed centrifuge tube containing the sample. The following parameter settings were applied: sampling time of 1 second per group, sensor self-cleaning duration of 100 seconds, sample preparation period of 5 seconds, injection flow rate of 400 ml/min, and sample analysis duration of 100 seconds.

##### 2.3.1.3 Sensor types

Various types of E-nose sensors exhibit strong responses to specific classes of characteristic gases during sample analysis. This sensitivity allows for the differentiation of primary volatile organic compounds (VOCs) present in the samples. This experimental E-nose instrument utilized in this study is equipped with 10 distinct metal oxide sensors. The specific aroma types corresponding to each of these sensors are comprehensively presented in **Table 2**.

**Table 2:** Electronic nose sensor arrays.

No.	Sensor Name	Sensitive substances
1	W1C	Aromatic compounds
2	W5S	Nitrogen oxides
3	W3C	Ammonia, aromatic compounds
4	W6S	Hydride
5	W5C	Alkanes aromatic compounds
6	W1S	Methane (methyl group)
7	W1W	Sulfides, terpenes
8	W2S	Alcohols
9	W2W	Organic sulfides
10	W3S	Alkanes, aliphatics

#### 2.3.2 GC-MS measurement

##### 2.3.2.1 Sample pre-treatment

Refer to 2.3.1.1 for sample treatment. To conduct the analysis, transferred a precisely measured quantity of the sample into a 15 ml headspace vial. Ensure a consistent headspace volume is maintained at the top of the vial. Subsequently, seal the vial with a cap equipped with an adhesive cushion, and securely fasten it to maintain sample integrity.

##### 2.3.2.2 Extraction processes

The extraction fiber head underwent aging according to the manufacturer’s instructions prior to use. This process involved exposing the head to a gasification chamber at 240°C for 30 minutes, after which it was removed to complete the aging procedure. For sample analysis, the headspace vial was placed in an electrically heated water bath. The extraction fiber head, attached to its handle, was then inserted into the sample vial. Using the handle, the exposed fiber was extended into the headspace for extraction. The system was equilibrated at 90°C for 40 minutes to facilitate the release of volatile substances. Following the completion of headspace extraction, the fiber was retracted, removed from the vial, and promptly inserted into the injection port of the gas chromatograph for desorption.

##### 2.3.2.3 GC-MS conditions

The volatile components were separated using an HP-5 MS capillary column (0.25 mm × 30 m, 0.25 µm). High-purity helium (>99.99%) served as the carrier gas, with a purge flow rate of 3.0 mL/min and an in-column gas flow rate of 1.78 mL/min. The system operated under pressure control at 100 kPa. The temperature ramp-up procedure followed the parameters outlined in **Table 3**. Chromatographic analysis was performed in full ion chromatography mode (m/z 35-550). The mass spectrometer utilized electron impact (EI) ionization with an energy of 70 eV and an ion source temperature of 230°C. Data acquisition was conducted in full-scan mode across a mass range of m/z 35-500, with the quadrupole temperature maintained at 150°C.

**Table 3:** Warming procedures.

	Rate (°C/min)	Final temperature (°C)	Retention time (min)
0	–	40	5
1	5	70	2
2	10	185	3
3	3	220	3
4	9	280	10

##### 2.3.2.4 Optimization of extraction conditions

Building upon previously optimized extraction conditions, a 50/30 μm DVB/CAR/PDMS solid-phase microextraction (SPME) fiber was employed. The extraction was conducted at 90°C for 40 minutes. To further enhance the extraction efficiency, critical parameters such as split ratio and injection volume were fine-tuned. The optimization process utilized an L_16_(4³) orthogonal experimental design, incorporating three factors (split ratio, injection volume, and hold time) at four levels each. The experimental levels were defined as follows: Level 1, splitless mode, 0.50 g injection volume, 2.00 min hold time; Level 2, 1:1 split ratio, 0.75 g injection volume, 2.50 min hold time; Level 3, 5:1 split ratio, 1.00 g injection volume, 3.00 min hold time; Level 4, 10:1 split ratio, 1.25 g injection volume, 3.50 min hold time. The design yielded a total of 16 unique treatment combinations, each replicated three times to ensure statistical robustness. **Table 4** presents a comprehensive overview of the various treatment levels employed in this study.

**Table 4:** Table of orthogonal design of extraction conditions.

No.	Process group	Split ratio		Injection volume		Resolution time	
		Level	Split ratio A	Level	Injection volume B(g)	Level	Resolution time C(min)
1	A2B2C4	2	1:1	2	0.75	4	3.50
2	A2B1C2	2	1:1	1	0.50	2	2.50
3	A4B1C4	4	10:1	1	0.50	4	3.50
4	A3B2C1	3	5:1	2	0.75	1	2.00
5	A3B1C3	3	5:1	1	0.50	3	3.00
6	A1B3C4	1	0	3	1.00	4	3.50
7	A1B1C1	1	0	1	0.50	1	2.00
8	A1B4C2	1	0	4	1.25	2	2.50
9	A3B4C4	3	5:1	4	1.25	4	3.50
10	A1B2C3	1	0	2	0.75	3	3.00
11	A4B2C2	4	10:1	2	0.75	2	2.50
12	A4B4C3	4	10:1	4	1.25	3	3.00
13	A2B4C1	2	1:1	4	1.25	1	2.00
14	A3B3C2	3	5:1	3	1.00	2	2.50
15	A4B3C1	4	10:1	3	1.00	1	2.00
16	A2B3C3	2	1:1	3	1.00	3	3.00

### 2.4 Data analysis

#### 2.4.1 Analysis of electronic nose data

Pattern recognition, which involves the computer-based processing and analysis of sensor output signals, plays a crucial role in the construction of the entire E-nose system (Miao et al., 2016). For this experiment, pattern recognition was conducted using the WinMuster platform. The analysis employed two methods: Linear Discriminant Factor Analysis (LDA) and Sensor Contribution Analysis (Loadings).

#### 2.4.2 Analysis of GC-MS data

The data were processed using GC-MS solution software (version 4.45, SHIMADZU, Kyoto, Japan). Retention indices of the isolated substances were calculated from their retention times and subsequently compared with the NIST 14 mass spectrometry database. Volatile components exhibiting matches greater than 80% were utilized as the basis for identification, with reference to the CAS number of each substance. For the qualitative analysis of volatile substances, the peak area normalization method was employed. The resulting statistical data were then input into SIMCA 14.1 software for Partial Least Squares Discriminant Analysis (PLS-DA) and Hierarchical Cluster Analysis (HCA).

## 3 Results

### 3.1 Electronic nose analysis

#### 3.1.1 Validation of electronic nose odor detection method

A sample was randomly selected and six groups of parallel tests were conducted to examine the repeatability of the method. The results showed that the response value RSD of each sensor was less than 5% (**Supplementary Table 1**), indicating that its repeatability was good.

A sample was randomly selected and tested at different time periods (0, 2, 4, 6, 8, 10, 12 h) to evaluate the stability of the sample. The results showed that the sample was relatively stable within 12 h, and the RSD was less than 5% (**Supplementary Table 2**).

#### 3.1.2 Electronic nose sensor response analysis

**Figure 2:**
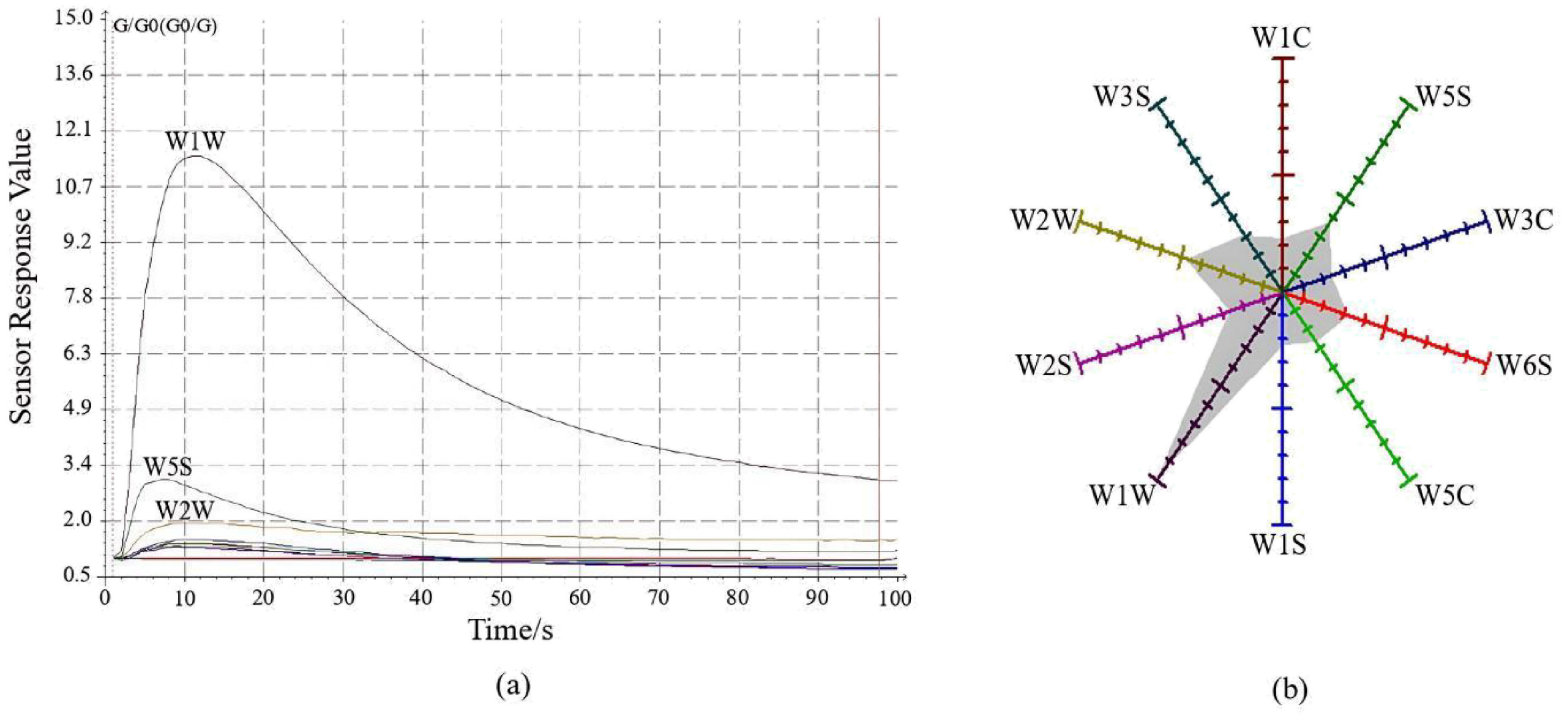
*Ligusticum chuanxiong* E-nose sensor characterization diagram. **(A)** Sensor signal response diagram; **(B)** Radar plot of response values at 98 s.

The electronic nose detection of the odors of CX and its medicinal relatives was conducted, as shown in **Figure 2A**. Upon exposure to the gas, the response values of the three sensors—W1W, W5S, and W2W—changed markedly, with a sharp increase peaking around 10 seconds. Subsequently, the response values sharply declined between 10 and 70 seconds, after which they stabilized until the end of the detection. The response values of each sensor at the 98th second in the steady state were used to create a radar plot (**Figure 2B**), where each sensor’s response value starts at 1 at the baseline and gradually increases outward. During the detection of CX and its medicinal relatives, sensors W1W, W5S, and W2W demonstrated higher responsiveness compared to other sensors. This suggests that these sensors are more sensitive to the specific chemical components present in the odors of CX and its medicinal relatives. The higher response could be related to the particular functional groups of the gas molecules, such as terpenes or sulfides, which interact more strongly with the sensor’s surface material. As indicated in **Table 5**, the resistance ratios of the 10 sensors varied across species, with sensor W1W exhibiting higher resistance ratios across all samples, suggesting that the odorants of CX and its medicinal relatives predominantly consist of terpenes and sulfides.

**Table 5:** Resistance ratios of CX and its medicinal relatives to 10 sensors in an electronic nose.

Sensor	CX	GSCX	YNCX	JCX	FX	GB	LGB
W1C	0.66 ± 0.16^e^	0.72 ± 0.03^c^	0.88 ± 0.14^b^	0.75 ± 0.10^b^	0.68 ± 0.03^fg^	0.74 ± 0.11^e^	0.76 ± 0.02^f^
W5S	1.15 ± 0.07^bc^	1.29 ± 0.12^b^	1.08 ± 0.05^ab^	1.09 ± 0.16^ab^	0.96 ± 0.06^c^	1.18 ± 0.10^bc^	0.98 ± 0.01^b^
W3C	0.68 ± 0.13^e^	0.72 ± 0.02^c^	0.88 ± 0.13^ab^	0.78 ± 0.10^b^	0.71 ± 0.03^ef^	0.74 ± 0.09^e^	0.80 ± 0.01^ef^
W6S	0.97 ± 0.01^c^	0.99 ± 0.01^bc^	1.01 ± 0.02^ab^	0.96 ± 0.02^ab^	0.99 ± 0.01^c^	0.98 ± 0.03^cd^	1.00 ± 0.02^b^
W5C	0.76 ± 0.11^de^	0.83 ± 0.02^c^	0.92 ± 0.09^b^	0.84 ± 0.07^b^	0.80 ± 0.02^d^	0.81 ± 0.07^de^	0.85 ± 0.01^cd^
W1S	0.65 ± 0.19^e^	0.65 ± 0.05^c^	0.95 ± 0.23^b^	0.75 ± 0.13^b^	0.63 ± 0.02^g^	0.76 ± 0.20^e^	0.81 ± 0.03^de^
W1W	2.52 ± 0.45^a^	3.88 ± 0.72^a^	1.73 ± 0.93^a^	1.81 ± 1.26^a^	1.25 ± 0.02^a^	2.65 ± 0.29^a^	1.09 ± 0.04^a^
W2S	0.72 ± 0.14^e^	0.71 ± 0.05^c^	0.95 ± 0.16^b^	0.81 ± 0.12^b^	0.74 ± 0.03^de^	0.80 ± 0.14^de^	0.89 ± 0.02^c^
W2W	1.33 ± 0.08^b^	1.34 ± 0.05^b^	1.11 ± 0.17^ab^	1.13 ± 0.22^ab^	1.07 ± 0.04^b^	1.31 ± 0.04^b^	1.00 ± 0.01^b^
W3S	0.95 ± 0.04^cd^	0.93 ± 0.01^c^	0.99 ± 0.06^b^	0.96 ± 0.01^ab^	0.93 ± 0.04^c^	0.98 ± 0.07^cd^	0.97 ± 0.01^b^

Based on analysis of variance (ANOVA), different lowercase letters in the same column indicate significant differences between sensors (*p*<0.05).

#### 3.1.3 Linear discriminant factor analysis

**Figure 3:**
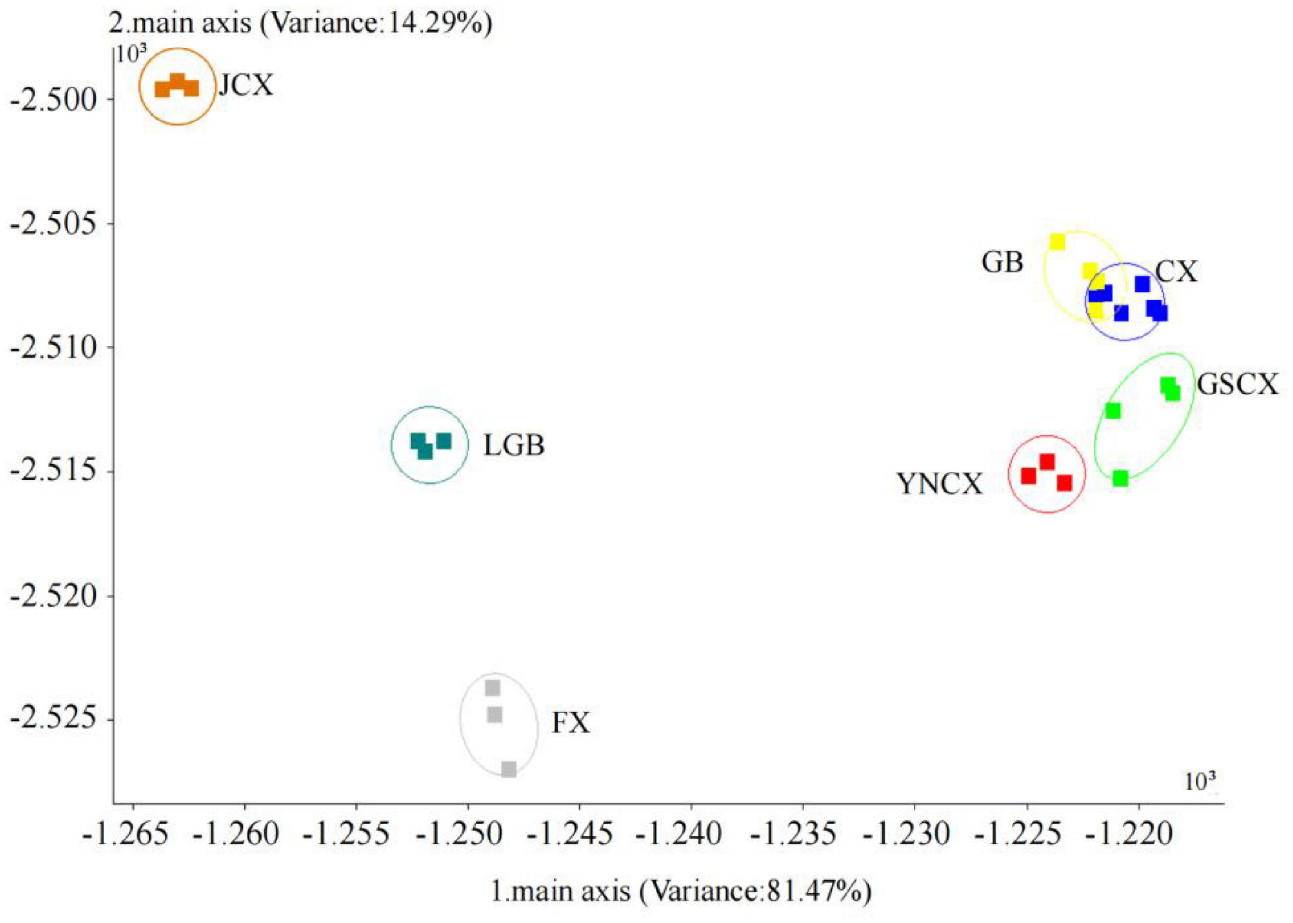
LDA map of *Ligusticum chuanxiong* and its medicinal relatives.

The odor characteristics of CX and its medicinal relatives were further analyzed using LDA, as illustrated in **Figure 3**. Linear discriminants LD1 and LD2 contributed 81.47% and 14.29%, respectively, explaining a total of 95.76% of the variance in the original variables. This indicates that the first two discriminants capture the majority of the variation in odor profiles across the samples, which suggests that the odors of CX and its medicinal relatives are highly distinguishable based on these two components. There was no overlap between the samples of JCX, LGB, FX, YNCX, and GSCX; they could all be completely separated. However, some samples of CX and GB still overlapped, suggesting that there might be similar volatile compounds between CX and GB, resulting in their indistinguishable odor signatures in the analysis. According to the LDA analysis, the E-nose can effectively distinguish the five closely related species of CX, highlighting its potential as a reliable tool for differentiating species with closely related odor profiles.

#### 3.1.4 Sensor contribution analysis (Loadings)

Loadings were utilized to analyze the response of the 10 metal sensors of the E-nose to the different types of volatiles in the samples (**Supplementary Figure 1**). PC1 contributed 92.36% and PC2 contributed 6.77%, totaling 99.13% of the variance. The correlation matrix pattern diagram shows that sensors W1W and W1S contribute more than 50% to PC1 and PC2, respectively. Along the X-axis, sensor W1W is furthest, suggesting that terpenes and sulfides contribute most to differentiation on the first principal component. Along the Y-axis, sensor W1S is furthest, indicating that methane has the greatest contribution to differentiation on the second principal component. Sensors W1C, W2S, W3C, and W5C also contribute to the second principal component. Sensors W5S, W3S, W6S, and W2W have low and negligible contributions to the first and second principal components. Terpenes, sulfides, methane components, aromatic compounds, alcohols, and alkane aromatic compounds were found to play important roles in distinguishing the odor characteristics of CX and its medicinal relatives using the E-nose technique.

### 3.2 HS-SPME-GC-MS analysis

#### 3.2.1 Methodological review

Precision test (**Supplementary Table 3**): Randomly select a sample as the material, repeat the injection 6 times, and calculate the total peak number and total peak area. It is found that in the 6 measurements, the peak number and area RSD are both less than 5%, indicating that the instrument precision is good.

Repeatability test (**Supplementary Table 4**): Randomly select a variety and inject 6 samples of the same variety as materials. The measured sample peak number and peak area RSD are both less than 5%, indicating that this method has good repeatability.

Stability test (**Supplementary Table 5**): Randomly select a sample as material and conduct tests at different time periods (0, 2, 4, 6, 8, 10, 12 h). It is found that the sample peak number and peak area RSD are all less than 5%, indicating that the experiment has good stability.

#### 3.2.2 Influence of extraction conditions on extraction effectiveness

In this experiment, Taguchi’s analysis was conducted on the total peak area and total peak number following the optimization of different extraction conditions. This analysis revealed how each factor’s level under various extraction conditions affected and influenced the total peak area and total peak number. **Supplementary Table 6**, the mean factor ranking for the total peak area was split ratio (A) > resolution time (C) > injection volume (B). The total peak area was maximized using A_1_B_3_C_1_, specifically a split ratio of 0, an injection volume of 1.00 g, and a resolution time of 2.00 min. **Supplementary Table 7** indicated that the mean factor ranking for the total number of peaks was split ratio (A) > resolution time (C) > injection volume (B). Using the A_1_B_3_C_1_ factor combination yielded the highest total number of peaks, enabling the detection of the maximum volatile components. The optimal combination derived from the analysis of both total peak area and total number of peaks was A_1_B_3_C_1_, indicating this combination as the optimal experimental condition for extraction.

#### 3.2.3 Types of volatile components

**Figure 4:**
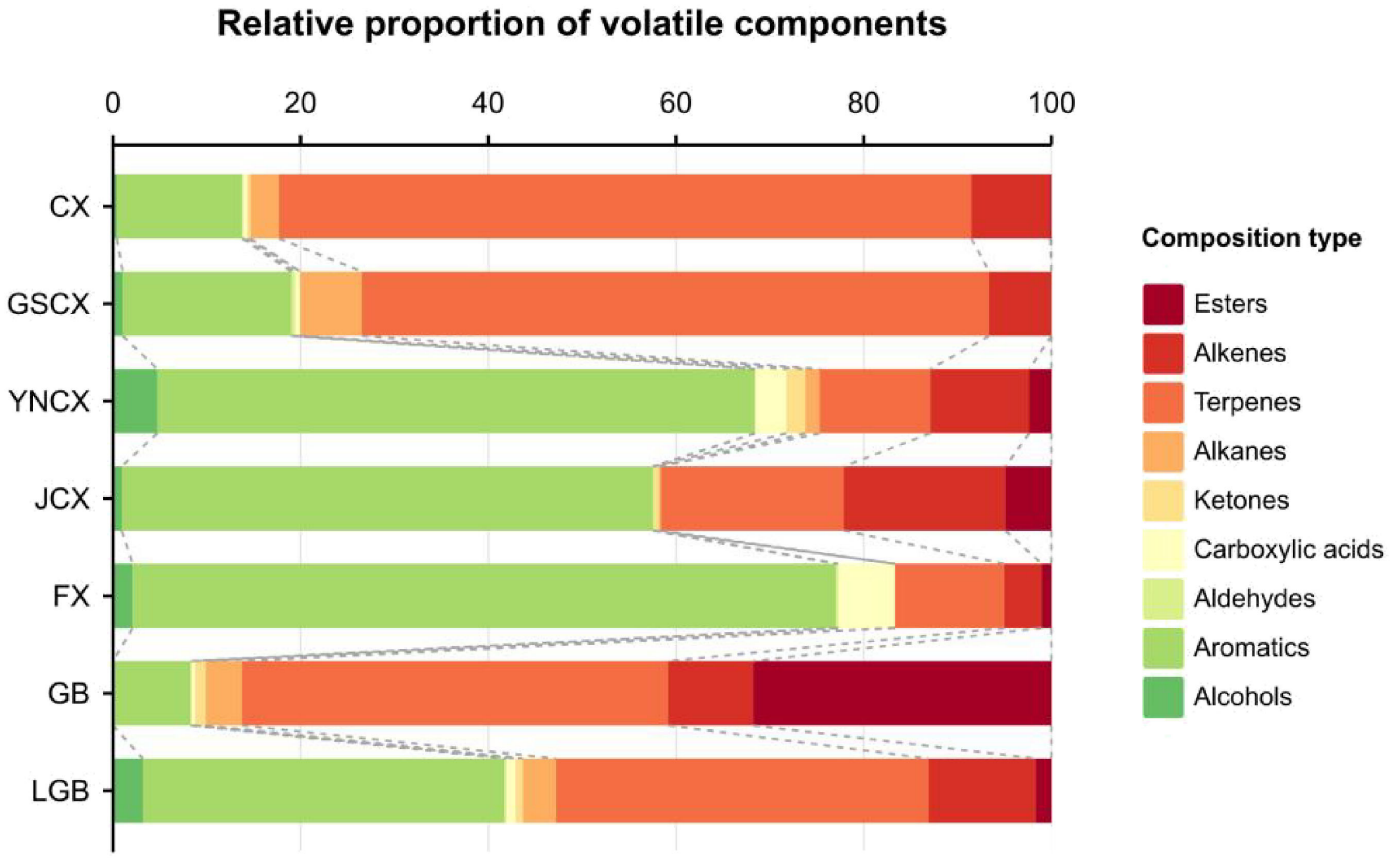
Volatile constituents of *Ligusticum Chuanxiong* and its medicinal relatives.

To investigate the volatile components of various medicinal species within the *Ligusticum* L., 27 sample sets were analyzed and identified using HS-SPME-GC-MS. **Table 6** shows the identification of 118 volatile components across CX, GSCX, YNCX, JCX, FX, GB, and LGB, encompassing 13 alcohols, 21 aromatics, 4 aldehydes, 9 carboxylic acids, 11 ketones, 6 alkanes, 29 terpenes, 14 esters, and 6 alkenes. YNCX had the highest number of volatile substances, with 55 identified, comprising 95.40% of the total volatile substances detected. Conversely, GB had the lowest number of volatiles, with 24 species identified, accounting for 88.63% of the total volatiles detected. According to **Figure 4**, CX, GSCX, GB, and LGB exhibited the highest proportions of terpenes, at 60.69%, 61.91%, 40.23%, and 36.88%, respectively. Conversely, YNCX, JCX, and FX showed the highest proportions of aromatics, at 60.77%, 51.26%, and 73.82%, respectively.

**Table 6:** Names and relative contents of volatile components of *Ligusticum chuanxiong* and its medicinal relatives.

NO.	Component^a^	Relative Content (%) ^b^						
		CX	GSCX	YNCX	JCX	FX	GB	LGB
	Alcohols							
1	Caryophyllene epoxide	0.09	0.21	–	0.37	0.87	–	–
2	Nerolidol	0.26	–	–	–	–	–	0.87
3	Isospathulenol	–	0.19	–	–	–	–	–
4	Viridiflorol	–	0.57	–	–	–	–	–
5	Lavandulo	–	–	–	0.12	–	–	–
6	α-cadinol	–	–	0.31	0.35	0.25	–	–
7	Phytol natural	–	–	–	–	0.12	–	–
8	Plantalcohol	–	–	1.64	–	0.85	–	1.29
9	α-Bisabolol	–	–	0.54	–	–	–	–
10	(2E,4S,7E)-1,7-dimethyl-4-(1-methylethyl)-2,7-Cyclodecadien-1-ol	–	–	1.63	–	–	–	0.67
11	β-bisabolol	–	–	–	–	–	–	0.13
12	Farnesol	–	–	0.24	–	–	–	–
13	Thumbergol	–	–	0.15	–	–	–	–
	Aromatics							
14	ϵ-Muurolene	0.17	0.15	–	–	–	–	0.17
15	(1S,4S,4aR)-1,2,3,4,4a,5,6,7-octahydro-4-methyl-7-methylene-1-(1-methylethyl)-Naphthalene	0.20	0.24	0.15	–	–	–	0.23
16	Hexahydro-3-butylphthalide	0.15	0.36	0.50	0.37	0.17	–	–
17	Butylphthalide	0.63	1.49	2.17	4.15	0.99	0.64	2.27
18	(E)-Ligustilide	5.93	0.35	1.85	1.84	0.44	–	16.22
19	trans-Neocnidilide	2.40	12.95	52.08	38.36	71.31	0.74	4.34
20	1,2,3,4,6,8alpha-Hexahydro-1-isopropyl-4,7-dimethylnaphthalene	0.15	–	–	–	–	–	–
21	4alpha-hydroxyendesm-11(13)-ene	0.12	–	–	0.28	0.07	–	–
22	3-Butylidenephthalide	1.24	–	–	–	–	–	–
23	Sesquirose furan	–	0.18	–	–	–	–	–
24	Senkyunolide	–	0.92	1.79	1.58	0.45	–	8.26
25	1,3,4-Eugenol methyl ether	–	–	–	0.36	–	–	–
26	4,7-dimethyl-1-propan-2-yl-1,2,3,5,6,8a-hexahydronaphthalene	–	–	–	0.89	–	–	–
27	(Z)-Butylidenephthalide	–	–	1.50	1.98	–	–	2.23
28	1,1,3-trimethyl-3-phenylindan	–	–	–	0.16	–	–	–
29	cis-Sedanolide	–	–	0.35	1.24	–	–	0.67
30	3-(4,8,12-Trimethyltridecyl)furan	–	–	–	0.05	0.10	–	–
31	n-Amylbenzene	–	–	–	–	0.29	–	–
32	Elemicin	–	–	–	–	–	6.01	–
33	Cnidimine	–	–	0.25	–	–	–	1.43
34	Diphenylamine	–	–	0.14	–	–	–	–
	Aldehydes							
35	3,5-Di-tert-butyl-4-hydroxybenzaldehyde	0.10	–	–	0.12	–	–	–
36	Farnesal	–	0.40	–	–	–	–	0.20
37	3-Heptylacrolein	–	–	–	–	0.07	–	–
38	2-Undecenal	–	–	–	–	0.19	–	–
	Carboxylic acids							
39	Citric acid	0.34	–	0.06	–	–	0.36	0.36
40	12-Hydroxystearic acid	–	0.14	–	–	–	–	–
41	2-Phenyl-2-ethylbutyric acid	–	0.36	0.71	–	–	–	–
42	Octanoic acid	–	–	–	–	0.37	–	0.10
43	Myristic acid	–	–	–	–	0.10	–	–
44	n-Hexadecanoic acid	–	–	2.31	–	4.32	–	0.44
45	Vaccenic acid	–	–	–	–	0.81	–	–
46	Stearic acid	–	–	–	–	0.28	–	–
47	3,7,11-Trimethyl-1,6,10-dodecatrien-3-yl-formic acid	–	–	0.11	–	–	–	–
	Ketones							
48	Pentanophenone	0.20	–	0.11	0.26	0.06	0.45	0.14
49	(E/Z)-Geranylacetone	0.08	–	–	–	–	–	–
50	β-Curcumene	–	–	–	0.24	–	–	0.44
51	2-(1-Cyclopent-1-enyl-1-methylethyl)cyclopentanone	–	–	–	–	–	0.23	–
52	salvial-4(14)-en-1-one	–	–	–	–	–	0.34	–
53	Hexahydrofarnesyl acetone	–	–	0.13	–	–	–	0.10
54	3,4-Dihydro-4,7,8-trimethylnaphthalen-1(2H)-one	–	–	–	–	–	–	0.07
55	4-Hydroxy-3-methylacetophenone	–	–	0.08	–	–	–	–
56	(E)-6,10-Dimethylundeca-5,9-dien-2-one	–	–	0.04	–	–	–	–
57	Camphostene	–	–	1.48	–	–	–	–
58	2-(5-oxohexyl)-Cyclopentanone	–	–	0.09	–	–	–	–
	Alkanes							
59	trans-3,6-diethyl-3,6-dimethyl-tricyclo[3.1.0.0(2,4)]hexane	2.22	6.10	1.22	–	–	3.43	3.10
60	1-Methyl-4-(1-methylethylidene)-2-(1-methylvinyl)-1-vinylcyclohexane	0.29	–	–	–	–	–	–
61	Heneicosanen	–	–	0.09	0.13	–	–	–
62	1-Methylene-4-(1-methylvinyl)cyclohexane	–	–	–	–	–	–	0.17
63	1-Chlorooctadecane	–	–	0.07	–	–	–	–
64	3,8-Dimethyldecane	–	–	0.08	–	–	–	–
	Terpenes							
65	α-Cubebene	0.43	0.30	0.06	0.09	–	–	0.12
66	β-Caryophyllene	17.93	35.77	2.65	3.32	0.72	7.65	7.75
67	(E)-β-Famesene	9.83	1.41	1.62	5.11	3.07	4.95	1.55
68	α-Caryophyllene	2.28	2.90	–	–	0.31	1.71	7.71
69	β-Selinene	20.40	0.85	0.83	3.64	4.23	4.84	0.53
70	γ-Muurolene	0.22	1.94	0.43	–	0.12	2.59	0.17
71	β-Elemene	2.19	–	0.24	0.85	0.13	1.17	–
72	α-Selinene	7.42	–	–	1.54	0.49	–	–
73	δ-Elemene	–	0.22	–	–	–	–	–
74	α-Bergamotene	–	11.07	–	0.60	0.83	–	11.57
75	α-Farnesene	–	6.98	1.73	–	–	–	3.27
76	γ-Cadinene	–	0.48	0.54	–	–	–	0.38
77	α-Curcumene	–	–	–	1.18	–	2.38	0.74
78	Calamenene	–	–	–	0.20	0.29	–	–
79	Myristicin	–	–	–	1.11	–	11.42	–
80	α-Copaene	–	–	–	–	0.40	–	–
81	Δ-Cadinene	–	–	–	–	0.71	–	–
82	α-Cadinene	–	–	0.15	–	0.06	–	–
83	α-Calacorene	–	–	–	–	0.13	–	–
84	β-Chamigrene	–	–	–	–	–	0.34	–
85	β-Bourbonene	–	–	–	–	–	–	0.35
86	Cadina-1,4-diene-cadinadiene	–	–	–	–	–	–	0.15
87	Cuparene	–	–	–	–	–	1.22	–
88	α-Pinene	–	–	–	–	–	–	1.15
89	β-Pinene	–	–	–	–	–	–	0.29
90	γ-Bisabolene	–	–	–	–	–	–	0.05
91	β-Cadinene	–	–	0.51	–	–	1.95	1.08
92	trans-α-Bergamotene	–	–	1.67	–	–	–	–
93	β-bisabolene	–	–	0.77	–	–	–	–
	Alkanes							
94	(E,E)-1,3,5-Undecatriene	0.64	2.67	1.98	4.64	1.69	–	0.19
95	5-Pentylcyclohexa-1,3-diene	0.82	0.42	3.98	2.28	–	–	0.83
96	Neophytadiene	5.18	2.46	2.74	7.64	2.10	5.52	7.10
97	1,3-Diisopropyl-1,3-cyclopentadiene	–	0.33	–	–	–	–	–
98	Oplopane	–	0.28	0.73	–	0.06	–	–
99	(1R,4R,5S)-1,8-dimethyl-4-(1-methylethenyl)-Spiro[4.5]dec-7-ene	–	–	–	0.15	–	0.23	0.09
100	1-(1,5-Dimethyl-4-hexen-1-yl)-4-methyl-1,3-cyclohexadiene	–	–	–	0.64	–	–	2.30
101	2,4-Diphenyl-4-methyl-1-pentene	–	–	0.07	0.24	–	–	–
102	1,4-Dimethyl-3-(2-methyl-1-propenyl)-4-vinyl-1-cycloheptene	–	–	–	–	–	2.27	–
103	Isoitalicene	–	–	–	–	–	–	0.11
104	1,5,9,9-Tetramethyl-1,4,7-cycloundecatriene	–	–	0.56	–	–	–	–
	Esters							
105	Lavandulyl acetate	0.10	–	0.21	0.40	0.09	1.03	–
106	R-Dihydroactinidiolide	–	–	0.41	0.15	–	–	0.33
107	Diethyl phthalate	–	–	–	0.69	0.10	–	–
108	Apioline	–	–	–	2.23	–	27.15	–
109	Methyl palmitate	–	–	–	0.23	–	–	–
110	Methyl linoleate	–	–	–	0.19	–	–	–
111	Bis(2-ethylhexyl) phthalate	–	–	0.19	0.55	–	–	0.07
112	Dibutyl phthalate	–	–	0.40	–	0.49	–	0.36
113	Glycidyl palmitate	–	–	–	–	0.19	–	–
114	Bis(6-methylheptyl) Phthalate	–	–	–	–	0.15	–	–
115	Bornyl acetate	–	–	–	–	–	–	0.80
116	1-Propylbenzoate	–	–	0.04	–	–	–	–
117	Methyl Linolenate	–	–	0.19	–	–	–	–
118	Bergapten	–	–	0.83	–	–	–	–

^a^Volatile components with MS match index >80%; ^b^Relative contents (%) = (Individual peak area/total peak area) × 100%. Peak areas were obtained by total ion chromatography (TIC) analysis. All data expressed as average.

#### 3.2.4 Analysis of major volatile components

**Table 7** illustrates that a few major volatile compounds constitute a significant portion of the total volatile constituents across different species, with the top five compounds accounting for 59.13%, 72.87%, 63.76%, 59.90%, 83.30%, and 57.75% of the total volatile constituents in CX, GSCX, YNCX, JCX, FX, GB, and LGB, respectively, totaling 51.51%. The major volatile constituents varied among species. In CX, β-Selinene constituted the highest percentage; GSCX showed β-Caryophyllene as the highest at 35.77%; YNCX, JCX, and FX exhibited trans-Neocnidilide as the highest at 52.08%, 38.36%, and 71.31%, respectively; Apioline constituted 27.15% as the primary volatile constituent in GB; (E)-Ligustilide was the predominant constituent in LGB and CX. Moreover, β-Selinene, β-Caryophyllene, and trans-Neocnidilide were common constituents among all seven medicinal plants.

**Table 7:** Top five volatile components in terms of relative abundance.

NO.	CX		GSCX		YNCX		JCX		FX		GB		LGB	
	Compounds	Ratio (%)	Compounds	Ratio (%)	Compounds	Ratio (%)	Compounds	Ratio (%)	Compounds	Ratio (%)	Compounds	Ratio (%)	Compounds	Ratio (%)
1	β-Selinene	20.40	β-Caryophyllene	35.77	trans-Neocnidilid	52.08	trans-Neocnidilid	38.36	trans-Neocnidilid	71.31	Apioline	27.15	(E)-Ligustilide	16.22
2	β-Caryophyllene	17.93	trans-Neocnidilide	12.95	5-Pentylcyclohexa-1,3-diene	3.98	Neophytadiene	7.64	β-Selinene	4.23	Myristicin	11.42	α-Bergamotene	11.57
3	(E)-β-Famesene	7.45	α-Bergamotene	11.07	Neophytadiene	2.74	(E)-β-Famesene	5.11	(E)-β-Famesene	3.36	β-Caryophyllene	7.65	Senkyunolide	8.26
4	α-Selinene	7.42	α-Farnesene	6.98	β-Caryophyllene	2.65	(E,E)-1,3,5-Undecatriene	4.64	Hexadecanoic acid	2.30	Elemicin	6.01	β-Caryophyllene	7.75
5	(E)-Ligustilide	5.93	trans-3,6-diethyl-3,6-dimethyl-Tricyclo[3.1.0.0(2,4)]hexane	6.10	Hexadecanoic acid	2.31	Butylphthalide	4.15	Neophytadiene	2.10	Neophytadiene	5.52	α-Caryophyllene	7.71

**Figure 5:**
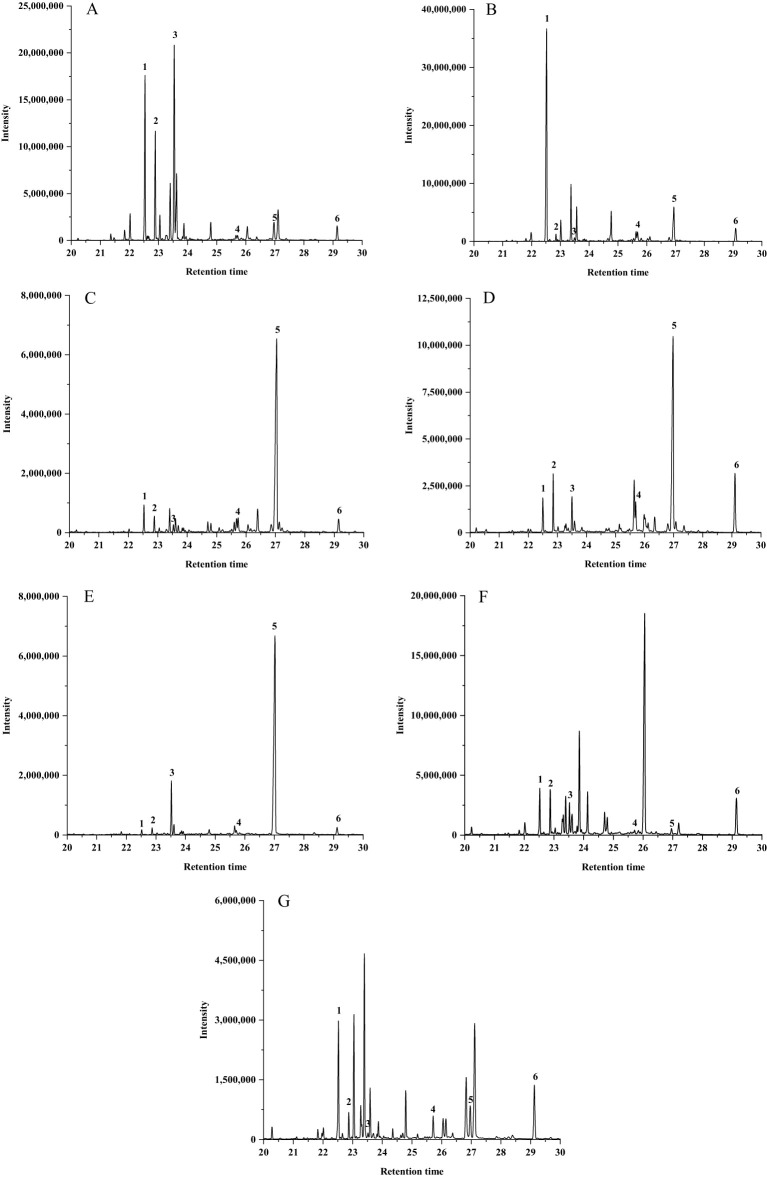
Chromatogram of peak intervals shared by *Ligusticum chuanxiong* and its medicinal relatives. **(A)** Chuanxiong (*Ligusticum chuanxiong*); **(B)** Gansu Chuanxiong (*Ligusticum chuanxiong* cv. Gansu); **(C)** Yunnan Chuanxiong (*Ligusticum chuanxiong* cv. Yunnan); **(D)** Japanese Chuanxiong (*Cnidium officinale* Makino); **(E)** Fuxiong (*Ligusticum sinense* ‘Fuxiong’); **(F)** Gaoben (*Ligusticum sinense*); **(G)** Liaogaoben (*Ligusticum jeholense*). 1: β-Caryophyllene; 2: (E)-β-Famesene; 3: β-Selinene; 4: Butylphthalide; 5: trans-Neocnidilide; 6: Neophytadiene.

Six volatiles were identified in CX and its medicinal relatives (**Figure 5**): peak 1 as β-Caryophyllene, peak 2 as (E)-β-Famesene, peak 3 as β-Selinene, peak 4 as Butylphthalide, peak 5 as trans-Neocnidilide, and peak 6 as Neophytadiene. The total relative contents of these six shared constituents in CX, GSCX, YNCX, JCX, FX, GB, and LGB were 55.45%, 54.92%, 62.08%, 62.23%, 75.91%, 25.54%, and 23.55%, respectively, indicating variations in their relative contents across different species.

#### 3.2.5 Characteristic volatile components analysis

**Figure 6:**
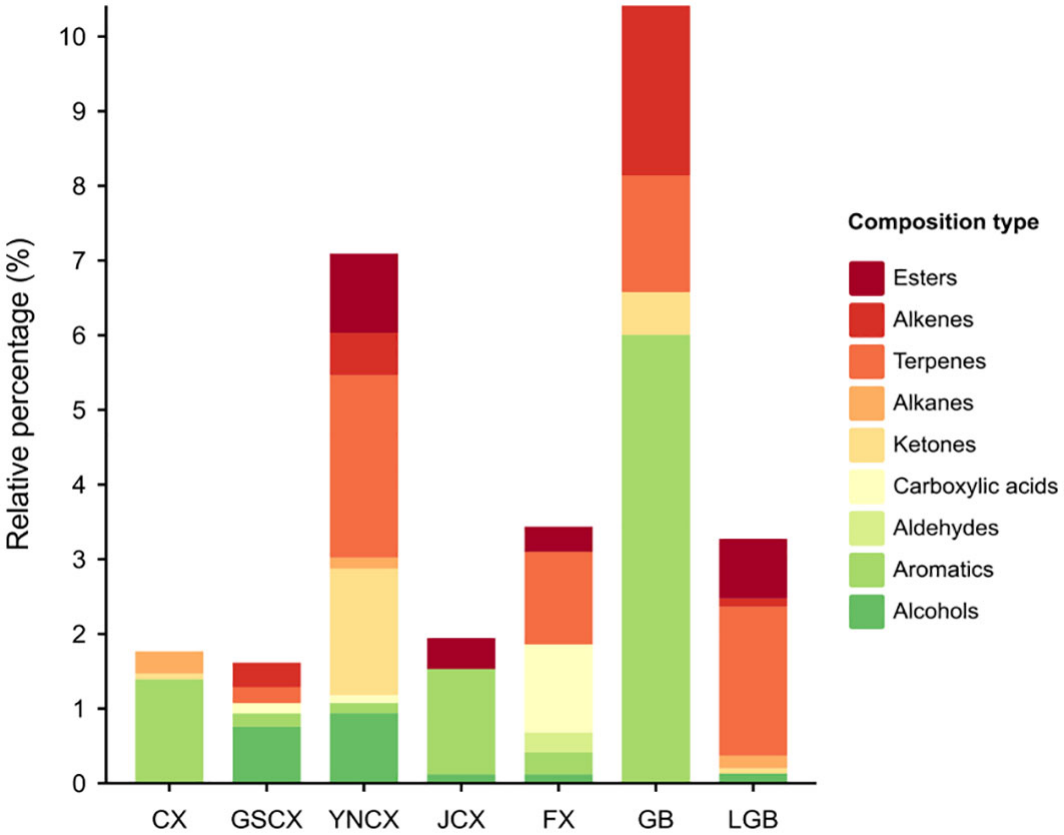
Types and relative contents of volatile components unique to *Ligusticum chuanxiong* and its medicinal relatives.

CX, GSCX, YNCX, JCX, FX, GB, and LGB each exhibited 4, 6, 17, 6, 12, 6, and 10 characteristic volatile components, constituting 1.77%, 1.61%, 7.09%, 1.94%, 3.44%, 10.41%, and 3.28% of the total components, respectively. **Figure 6** illustrates that CX’s characteristic volatile components include aromatics, ketones, and alkanes, with 3-Butylidenephthalide having the highest relative content at 1.24%. GSCX’s components encompass alcohols, aromatics, carboxylic acids, terpenes, and alkenes, with Viridiflorol leading at 0.57%. YNCX features a diverse range of alcohols, aromatics, acids, ketones, alkanes, terpenes, alkenes, and esters, including trans-α-Bergamotene at 1.67%. JCX includes an alcohol, three aromatics, and two esters, with 4,7-dimethyl-1-propan-2-yl-1,2,3,5,6,8a-hexahydronaphthalene reaching 0.89%. FX’s profile includes alcohols, aromatics, aldehydes, acids, terpenes, and esters, led by Vaccenic acid at 0.81%. GB comprises an aromatic, two ketones, two terpenes, and an alkene, with Elemicin at 6.01%. LGB boasts the most terpenes, totaling five at 2.00%, with α-pinene reaching 1.15%. Aromatic compounds dominate CX, JCX, and GB, while terpenes prevail in YNCX, FX, and LGB.

#### 3.2.6 Partial least squares discriminant analysis

**Figure 7:**
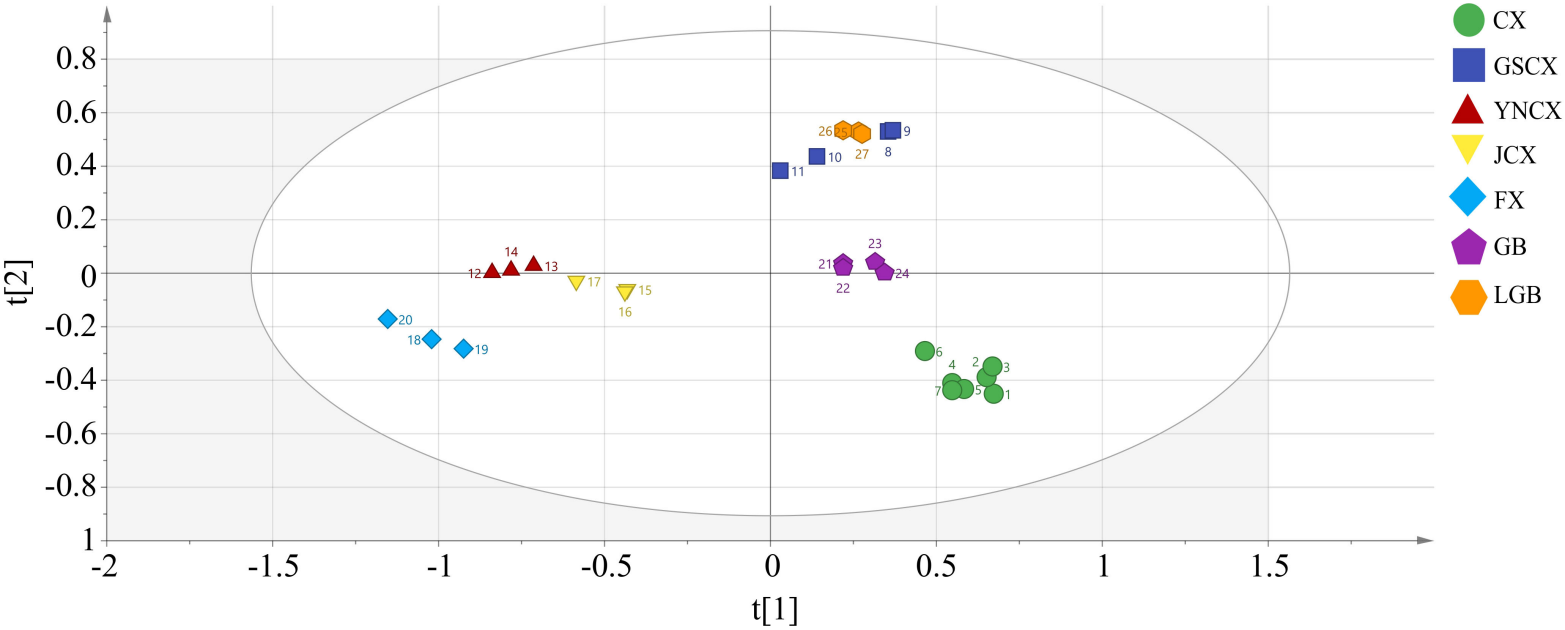
Plot of PLS-DA scores for volatile components of leaves.

In order to investigate further the relationship among the volatile components of CX and its medicinal relatives, sample categories were predicted using a PLS-DA model. R2X=0.971, R2Y=0.968, Q2 = 0.848 in the established model (**Figure 7**), indicating that the model can well reduce the dimensionality of this experimental data and has good accuracy. **Supplementary Figure 2** demonstrates that the model, tested with 200 permutations, consistently showed original R2 and Q2 values higher than permuted values, confirming its lack of overfitting, stability, and suitability for exploring differential volatile constituents of CX and closely related medicinal species. The five samples can be completely separated in the PLS-DA model, but there is overlap between the samples of LGB and GSCX. Therefore, the OPLS-DA model can be further used to distinguish the samples of LGB and GSCX.

**Figure 8:**
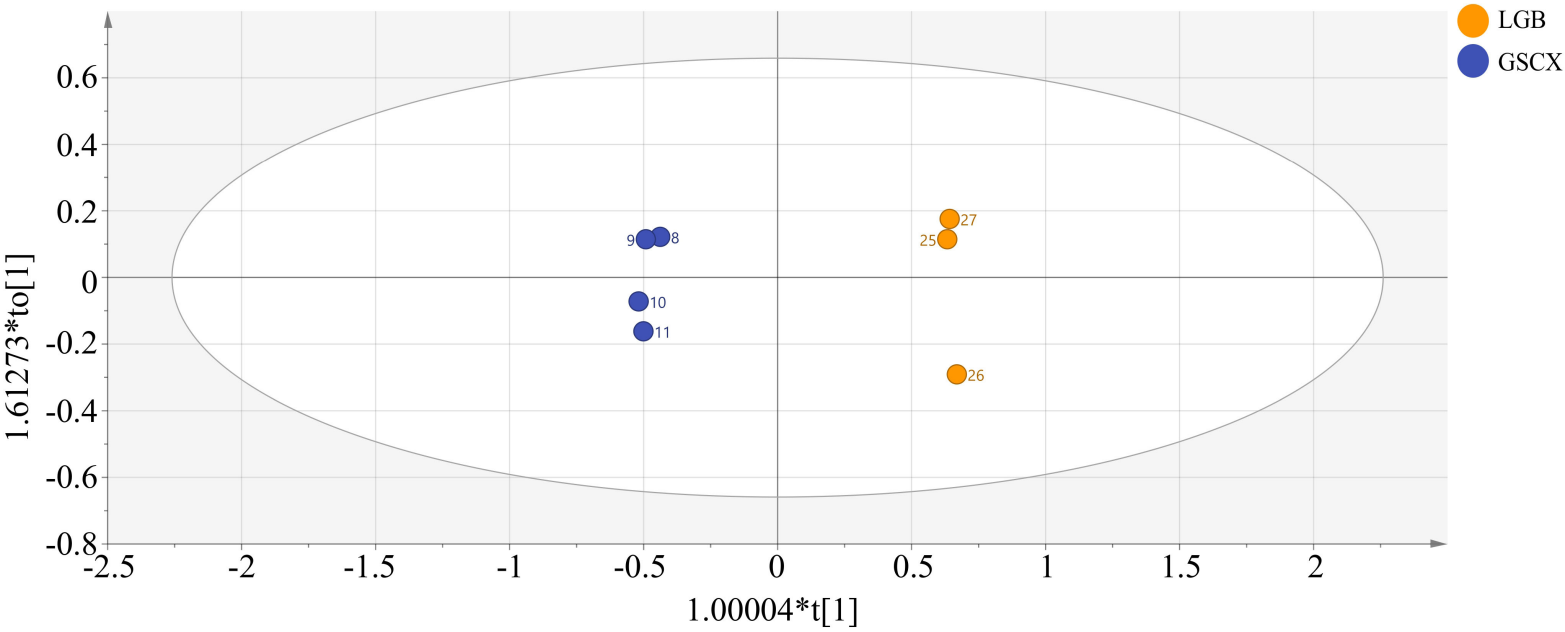
Plot of OPLS-DA scores for volatile components of leaves.

As shown in **Figure 8**, an OPLS-DA model was established between the samples of LGB and GSCX. It was found that the model can completely distinguish the two groups of samples. In the model, R2X=0.912, R2Y=0.998, Q2 = 0.995, and after permutation test There is no overfitting phenomenon (**Supplementary Figure 3**), indicating that this model is suitable for exploring the differential volatile components between LGB and GSCX samples.

#### 3.2.7 Screening for differential volatile components

Variable Important for the Projection (VIP) is used to indicate the strength of influence and explanatory power of volatile components in categorically discriminating groups of samples. It aids in screening key differential components (Jin et al., 2024). As shown in **Supplementary Figures 4**, **5**, with *P*<0.05 and VIP>1 as the standards, a total of 8 differential volatile substances were screened out through the model established in 3.2.6, including aromatic, terpenoid, and alkene, trans-Neocnidilide, β-Caryophyllene, β-Selinene, 5-pentylcyclohex-1,3-diene, (E)-Ligustilide, Butylphthalide, Neophytadiene, and Senkyunolide are key compounds that differ in the volatile components of CX and its medicinal relatives.

**Figure 9:**
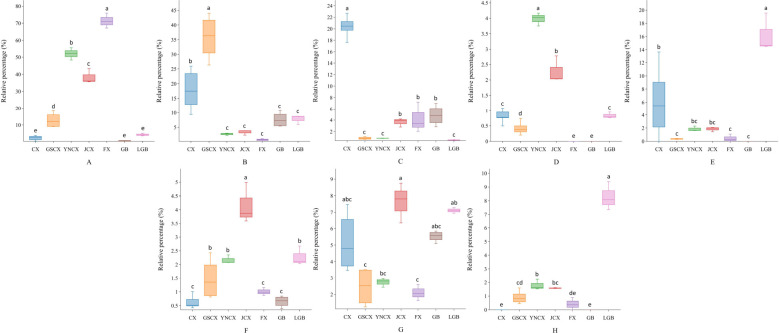
Differential volatile component box plots. **(A)** trans-Neocnidilide; **(B)** β-Caryophyllene; **(C)** β-Selinene; **(D)** 5-Pentylcyclohexa-1,3-diene; **(E)** (E)-Ligustilide; **(F)** Butylphthalide; **(G)** Neophytadiene; **(H)** Senkyunolide. Lowercase letters indicate significant differences between different species (P<0.05).

The comparison of the relative contents of the screened differentially volatile constituents revealed varying degrees of variation among the species. As shown in **Figure 9**, in CX leaves, β-Selinene exhibited significantly higher relative content compared to GSCX, YNCX, JCX, FX, GB, and LGB. β-Caryophyllene showed significantly higher relative content in GSCX compared to the other species. 5-pentylcyclohex-1,3-diene in YNCX was significantly different from the other species. trans-Neocnidilide exhibited significantly higher content in FX compared to the other closely related species. The relative contents of Butylphthalide and Neophytadiene in JCX were both high, and a significant difference was observed between Butylphthalide and the other species. LGB was significantly distinguished from the other closely related species by its use of (E)-Ligustilide and Senkyunolide.

#### 3.2.8 Cluster analysis of volatile components

**Figure 10:**
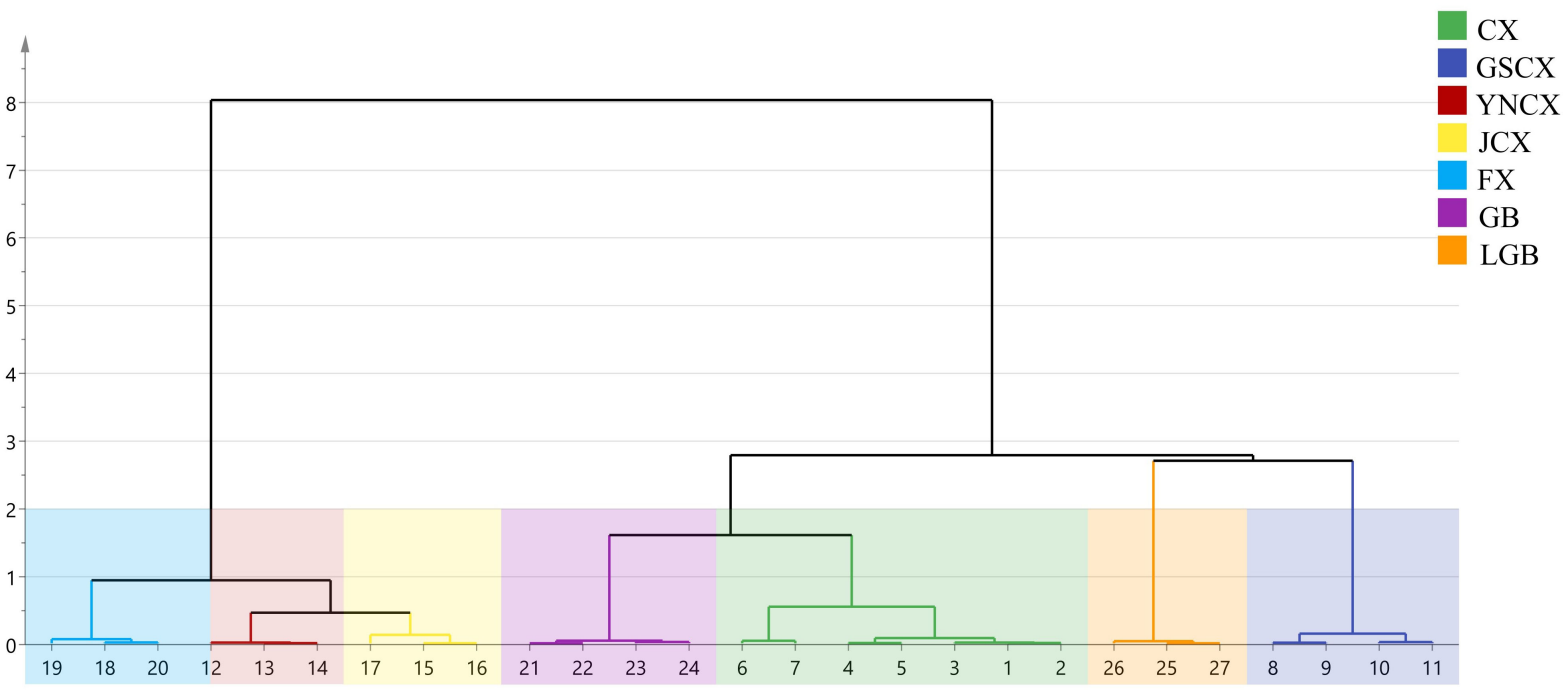
Hierarchical cluster analysis (HCA) of volatile components.

The volatile components of CX and its medicinal relatives were clustered and analyzed. As depicted in **Figure 10**, all samples were categorized into two groups: CX, GB, LGB, and GSCX clustered together in one category, while FX, YNCX, and JCX clustered in another. This clustering suggests that the volatile profiles of CX, GB, LGB, and GSCX are more similar to each other, indicating that these species may share a common chemical or metabolic pathway for producing their characteristic odorants. The analysis revealed variations in volatile components among these herbs, with specific differences in the types and concentrations of volatile compounds contributing to the distinct clustering patterns. These findings suggest closer affinities between CX and GB, GSCX and LGB, and YNCX and JCX. Such affinities may be related to their genetic relationships, environmental factors, or shared plant metabolic pathways that influence the production of specific volatiles.

### 3.3 Correlation analysis

**Figure 11:**
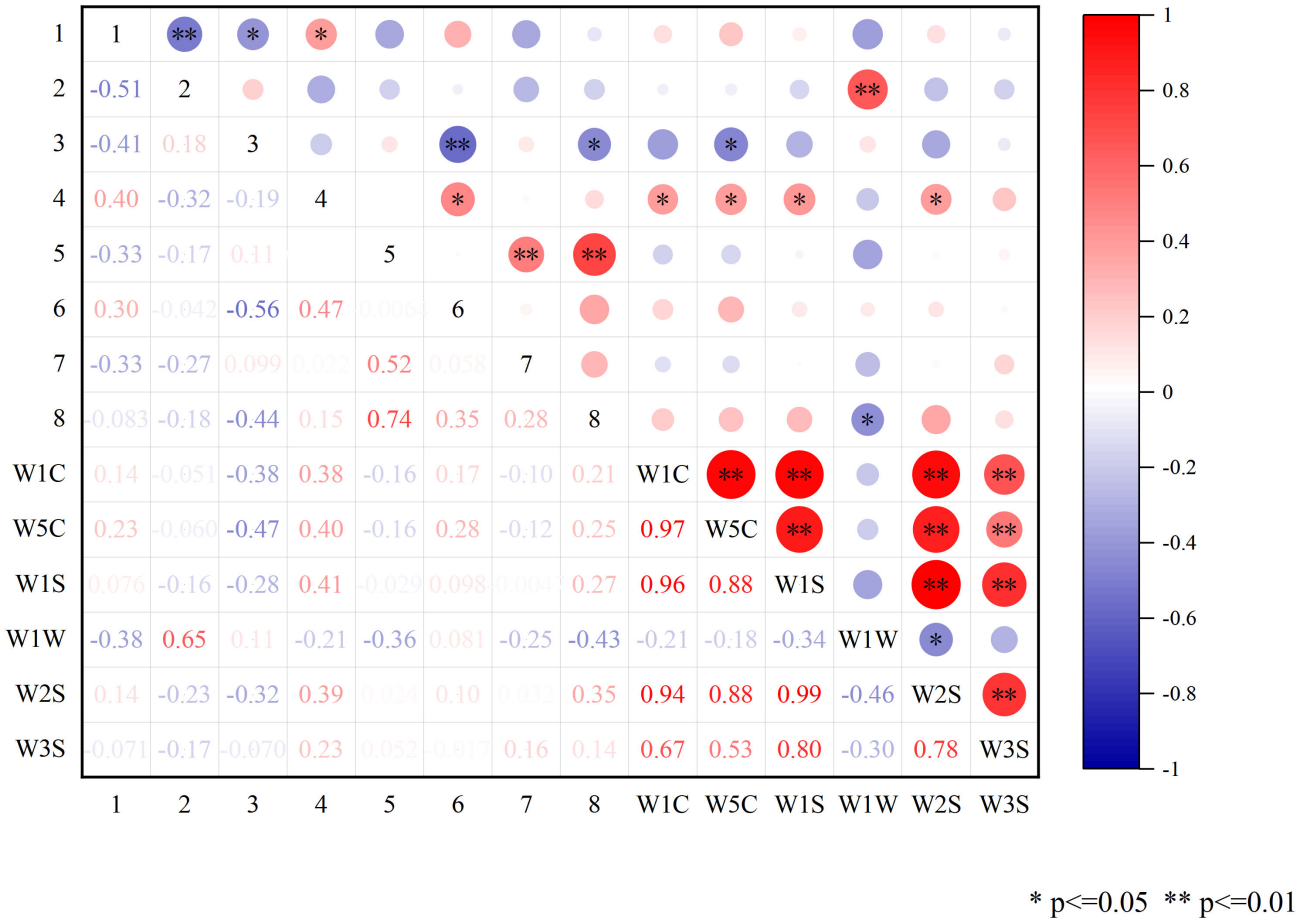
Pearson correlation analysis of differential components with E-nose sensors. Red represents positive correlation, blue represents negative correlation, white is non-significant correlation, and the darker the color in the graph, the stronger the correlation; 1: trans-Neocnidilide; 2: β-Caryophyllene; 3: β-Selinene; 4: (E)-Ligustilide.

To explore the intrinsic relationship between volatile components and odor in CX and its medicinal relatives, Pearson correlation analysis was conducted between the differential volatile components and the contributing E-nose sensors (**Figure 11**). The results showed that there were crossovers in compound types between some sensors. Specifically, certain volatile compounds exhibited strong correlations with particular sensors, suggesting that specific sensor types are more sensitive to certain chemical classes. Among them, β-Caryophyllene and Senkyunolide had significant correlations with the sensor W1W. β-Caryophyllene showed a very significant positive correlation with W1W, while Senkyunolide showed a significant negative correlation. This suggests that terpenes may play a crucial role in distinguishing CX and its medicinal relatives, with β-Caryophyllene potentially being a key component. The contrasting correlations of β-Caryophyllene and Senkyunolide with W1W highlight the diversity of volatile compounds that contribute to the odor profile and further support the idea that E-nose sensors can selectively respond to different compound types, reflecting their varying chemical properties.

## 4 Discussion and conclusions

In traditional taxonomy, CX, GSCX, YNCX, and FX are closely related, collectively referred to as ‘Xiongqiong’ due to their different origins (Li, 2005). Comparative analysis of the volatile constituents of the rhizomes of CX, JCX, FX, and JX (YNCX) revealed that CX is more similar to JCX, while FX is closer to JX (Huang et al., 2023). In this study, FX was found to be closer to YNCX and JCX, both of which were introduced from CX, likely differing due to geographical and anthropogenic factors. The results of the E-nose and GC-MS measurements indicated that CX was most similar to GB. CX is recognized as a cultivated variant of the congener GB, named *L. sinense* cv. chuanxiong in FRPS (Pu, 1991), and no wild species of CX have been reported. The findings of modern sporology (Wang, 1990) and chromosomal karyotyping (Fang and Zhang, 1984) studies also support the close affinity between CX and GB.

Previous studies have indicated that *Ligusticum* L. is not a monophyletic taxon (Katz-Downie et al., 1999; Zhou et al., 2008; Ren et al., 2020), and the genomic cross-hybridization between *C. officinale* and GB has suggested that CX may also have originated from *C. officinale* (Lee et al., 2010). Phylogenetic inference using plastid genomic data revealed that CX is more closely related to LGB than to GB (Yuan et al., 2021). The haplotype genome analysis indicated that CX is a sterile hybrid derived from two different diploid parents, with GB as one parent, and the other potential parent could be LGB, *C. officinale*, or *Ligusticum nematophyllum* (Nie et al., 2024). In traditional medicine, the dried rhizomes of GB and LGB have similar efficacy and morphology, making it difficult to differentiate and characterize them using DNA barcoding and ITS2 (Wei et al., 2022), suggesting a very close relationship. Clustering analysis of the volatile components of the leaves revealed that the samples were divided into two branches: one consisting of CX, GB, LGB, and GSCX. This indicates that their volatile components are closely related, confirming that CX is very close to GB and LGB, and that the three can be significantly differentiated using β-Selinene and (E)-Ligustilide. ‘Xixiong’ was once thought to have originated from GB (Shan and Hao, 2011) and it was called by the name CX. However, in this study, GSCX (Xixiong) was found to be closest to LGB, suggesting that it may also be a hybrid with parents from GB and LGB. This supports the hypothesis that another potential parent of CX could have been LGB. Future studies could combine LC-MS and GC-IMS to conduct correlation analysis on targeted metabolomics or non-volatile components, deeply exploring the mechanisms of action of the different components of CX and its closely related species, and further investigating their interspecies relationships.

Many traditional medicinal herbs have distinctive odors (Xu et al., 2011). In previous studies, the main types of substances in the volatile oil of CX leaves were found to be similar to those of the rhizomes, with the main constituents including (E)-Ligustilide, trans-Neocnidilide, and β-Selinene, though the (E)-Ligustilide content was lower in the leaves than in the rhizomes (Liu et al., 2020; Zhang et al., 2021). The results of the present experiment were similar to those of previous studies in terms of the types of constituents identified (Zhang et al., 2019), but the contents varied, likely due to differences in the environment of the sampling site and sample pre-treatment. Many terpenes have been serendipitously discovered in medicine and are commonly used as signaling molecules (Hillier and Lathe, 2019). They may also possess numerous medicinal properties (Guo et al., 2019; Salminen et al., 2008). The E-nose and GC-MS assays revealed that terpenes accounted for a significant proportion of the sample composition and played a crucial role in distinguishing CX and its medicinally related species. As a key constituent, β-Caryophyllene, a medicinal sesquiterpene with an odor intermediate between clove and turpentine, belongs to the cannabis terpenoids along with limonene, α-pinene, caryophyllene oxide, and phytol. These terpenoids have been shown to significantly inhibit *Pseudomonas syringae* in Solanaceae and Arabidopsis thaliana plants (Russo, 2011). As a ligand for cannabinoid receptor 2 (CB2), it also had the ability to improve wound healing (Koyama et al., 2019). The synthesis of β-caryophyllene mainly relied on isoprene and was catalyzed by terpene synthase. When plants were under environmental stress, phytohormones (such as jasmonic acid, ethylene, etc.) could further regulate the expression of terpene synthase genes, thereby increasing the biosynthesis of β-caryophyllene. The highest percentage component of CX leaves, β-Selinene, is a known antifungal compound that can be induced by jasmonic acid in *Apium graveolens* L (Stanjek et al., 1997). It is also believed to inhibit insect feeding and pollination and can be used as a pollinator-mediated attraction regulator in agricultural environments (Quarrell et al., 2023). However, the proportion of β-Selinene in GB and LGB is low, suggesting that the leaf odor inhibiting insect pollination may also contribute to reduced fruit set. Trans-Neocnidilide, (E)-Ligustilide, and Senkyunolide, all present in significant amounts among different species, effectively inhibit the production of nitric oxide in hepatocytes (Ningsih et al., 2020) and the signaling of inflammatory factors (Ma et al., 2018), forming the basis for their anti-inflammatory efficacy. It was found that CX rhizome stems and leaves contain more phthalide dimers and trimers, which exhibit greater antioxidant activity compared to other parts of the plant (Yan et al., 2022). In this experiment, Butylphthalide and Neophytadiene were found to be present in all seven species, suggesting their potential as anti-inflammatory and immunomodulatory therapies, as well as their involvement in anti-anxiety and anticonvulsant activities (Zhang et al., 2022; Gonzalez-Rivera et al., 2023). At present, the exact biosynthetic mechanism of Butylphthalide is not clear, but some studies have shown that Senkyunolide A can be converted into Butylphthalide under the catalysis of LcSAO1, and the three amino acid residues T98, S176, and T178 play a key role in substrate binding and enzyme activity (Chen et al., 2023). Therefore, the leaves of CX and its related species also possess high medicinal value and hold the potential to expand medicinal sources. Their efficacy and mechanisms of action require further study.

## Data Availability

The raw data supporting the conclusions of this article will be made available by the authors, without undue reservation.

## Supplementary material

Supplementary Figure 1 E-nose sensor Loadings analysis of volatile components in leaves of *Ligusticum chuanxiong* and its medicinal relatives.

Supplementary Figure 2 PLS-DA replacement test results.

Supplementary Figure 3 OPLS-DA replacement test results.

Supplementary Figure 4 PLS-DA model VIP diagram.

Supplementary Figure 5 OPLS-DA model VIP diagram.

Supplementary Table 1 Electronic nose detection repeatability test results.

Supplementary Table 2 Electronic nose detection stability test results.

Supplementary Table 3 GC-MS precision test results.

Supplementary Table 4 GC-MS repeatability test results.

Supplementary Table 5 GC-MS stability test results.

Supplementary Table 6 Total peak area mean response table.

Supplementary Table 7 Total peak number mean response table.
