# Supplementary material for: E-Nose and HS-SPME-GC-MS unveiling the scent signature of Ligusticum chuanxiong and its medicinal relatives
Source: Front Plant Sci. 2025 Mar 10;16:1476810. doi: 10.3389/fpls.2025.1476810 (PMC11931069; doi:10.3389/fpls.2025.1476810)
Supplement: Supplementary Figure 1 — E-nose sensor Loadings analysis of volatile components in leaves of Ligusticum chuanxiong and its medicinal relatives. [file SupplementaryFile1.pdf]

Figures

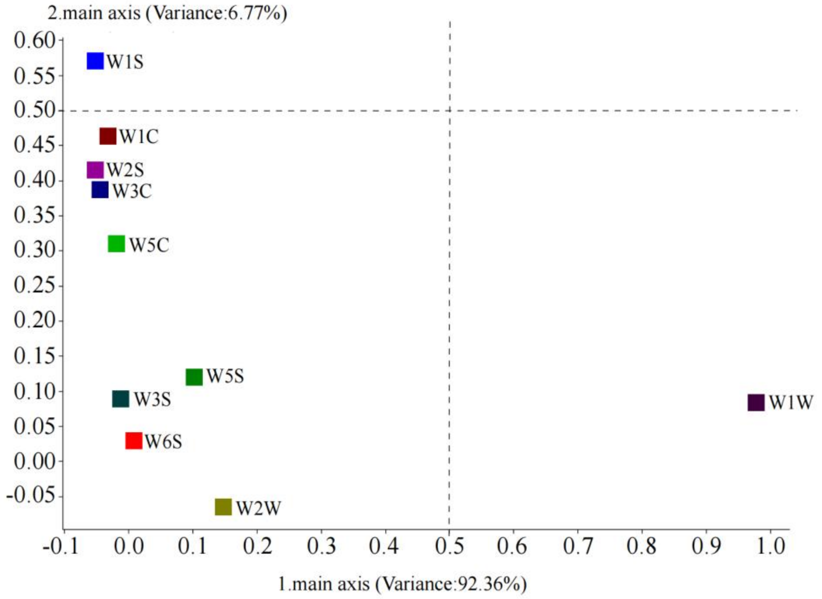

FIGURE 1 E-nose sensor Loadings analysis of volatile components in leaves of *Ligusticum chuanxiong* and its medicinal relatives.

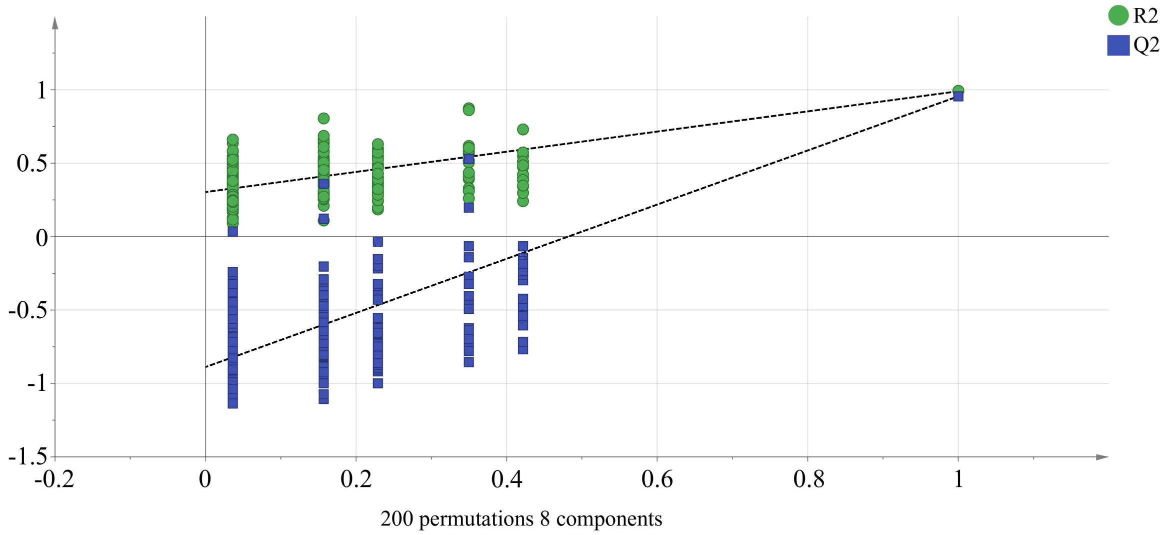

FIGURE 2 PLS-DA replacement test result

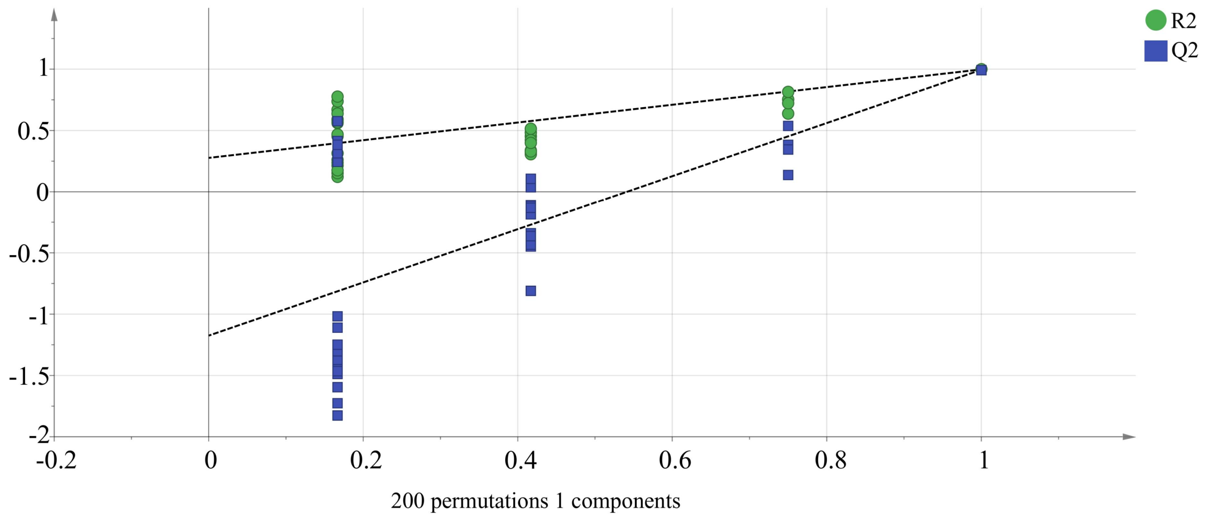

FIGURE 3 OPLS-DA replacement test results.

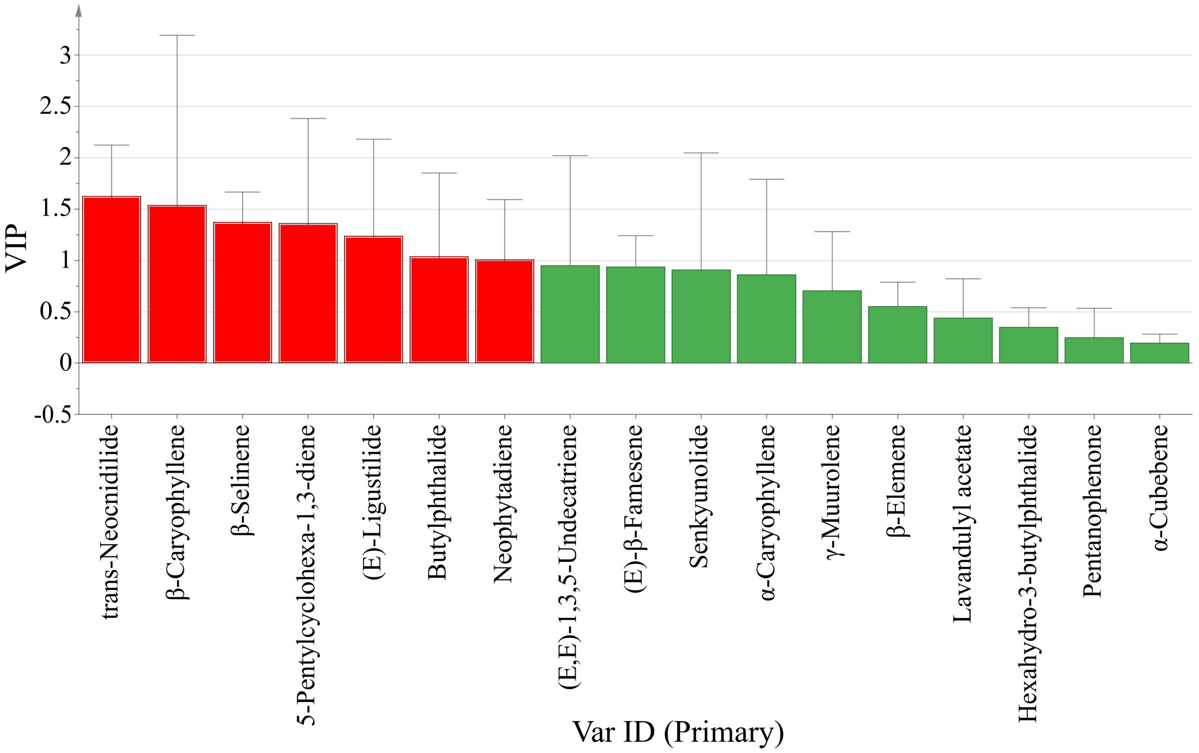

FIGURE 4 PLS-DA model VIP diagram.

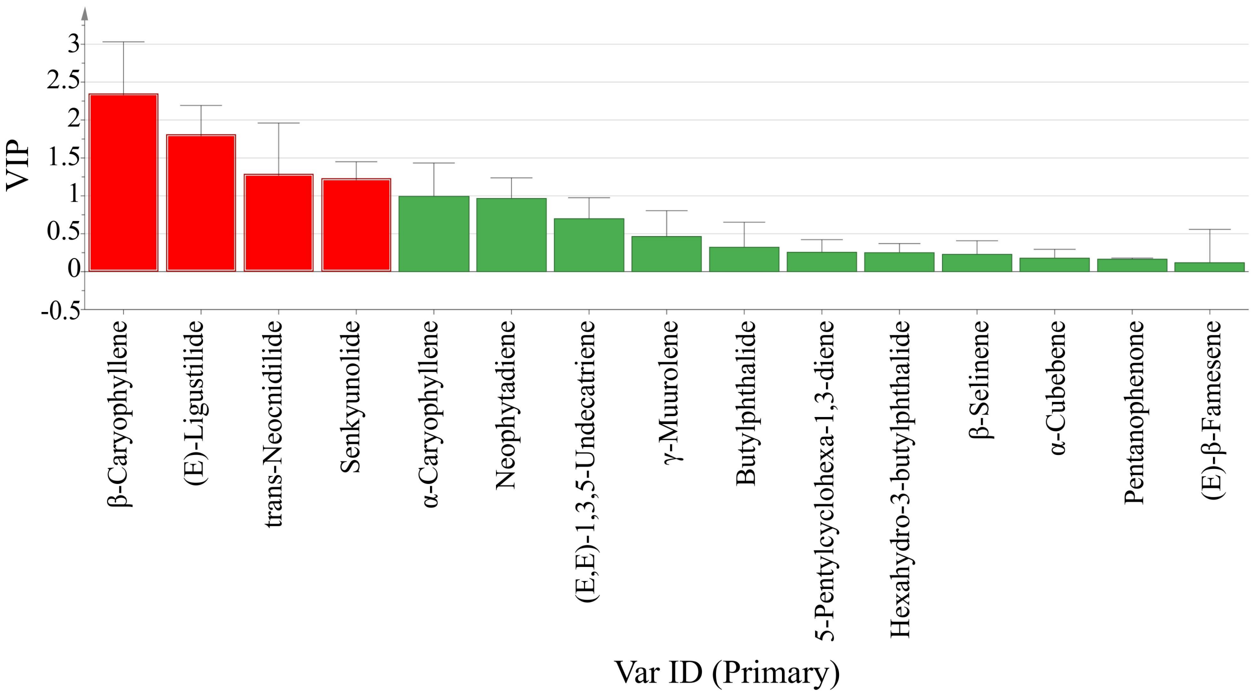

FIGURE 5 OPLS-DA model VIP diagram.

Tables

TABLE 1 Electronic nose detection repeatability test results.

Sensor	Sensor response						RSD (%)
	1	2	3	4	5	6	
W1C	0.710	0.710	0.713	0.708	0.709	0.710	0.213
W5S	2.066	2.057	2.050	2.073	2.068	2.063	0.390
W3C	0.701	0.702	0.703	0.702	0.703	0.704	0.149
W6S	1.380	1.378	1.378	1.396	1.398	1.396	0.726
W5C	0.699	0.699	0.700	0.702	0.703	0.703	0.283
W1S	1.290	1.290	1.288	1.267	1.264	1.262	1.069
W1W	11.995	11.940	11.885	12.778	12.746	12.694	3.566
W2S	1.138	1.137	1.136	1.129	1.129	1.128	0.417
W2W	1.013	1.019	1.015	1.009	1.016	1.011	0.348
W3S	0.944	0.942	0.943	0.964	0.967	0.968	1.352

TABLE 2 Electronic nose detection stability test results.

Sensor	Sensor response							RSD（%）
	0 h	2 h	4 h	6 h	8 h	10 h	12h	
W1C	0.652	0.672	0.673	0.673	0.664	0.665	0.665	1.128

W5S	2.551	2.543	2.534	2.348	2.489	2.479	2.469	2.802
W3C	0.634	0.635	0.635	0.674	0.658	0.659	0.660	2.444
W6S	1.491	1.490	1.489	1.485	1.488	1.485	1.451	0.944
W5C	0.644	0.645	0.645	0.660	0.644	0.646	0.646	0.868
W1S	1.415	1.336	1.334	1.332	1.334	1.333	1.330	2.319
W1W	16.441	16.372	16.302	15.751	15.731	15.691	14.503	4.227
W2S	1.249	1.182	1.180	1.181	1.175	1.174	1.172	2.294
W2W	1.012	1.017	1.011	1.018	1.016	1.013	1.017	0.273
W3S	0.962	0.963	0.999	0.998	0.973	0.973	0.973	1.565

TABLE 3 GC-MS precision test results.

Type	Test number						RSD (%)
	1	2	3	4	5	6	
Total number of peaks	52	51	51	50	51	49	2.04
Total peak area	23415618	21561123	21326215	21034719	22343568	22424584	4.00

TABLE 4 GC-MS repeatability test results.

Type	Test number						RSD (%)
	1	2	3	4	5	6	
Total number of peaks	44	43	42	44	46	45	3.21
Total peak area	30456288	29241876	28792416	30030996	32347495	31266206	4.32

TABLE 5 GC-MS stability test results.

Type	Time (h)							RSD (%)
	0	2	4	6	8	10	12	
Total number of peaks	50	51	51	53	54	54	50	3.42
Total peak area	162280160	161379156	165224680	171878821	178957135	170209134	155169242	4.73

TABLE 6 Total Peak Area Mean Response Table.

Level	Split ratio	Injection volume	Resolution time
1	28624624	13383596	15328011
2	14904928	12180403	12500635
3	4191600	14867231	14617187
4	5594898	12884819	10870217
Delta	24433024	2686828	4457795
Rank	1	3	2

TABLE 7 Total Peak Number Mean Response Table.

Level	Split ratio	Injection volume	Resolution time
1	48	25.25	29.5
2	30.75	27.00	28.5
3	16	29.75	28
4	16	28.75	24.75
Delta	32	4.50	4.75
Rank	1	3	2
